# Structural resolution of switchable states of a de novo peptide assembly

**DOI:** 10.1038/s41467-021-21851-8

**Published:** 2021-03-09

**Authors:** William M. Dawson, Eric J. M. Lang, Guto G. Rhys, Kathryn L. Shelley, Christopher Williams, R. Leo Brady, Matthew P. Crump, Adrian J. Mulholland, Derek N. Woolfson

**Affiliations:** 1 School of Chemistry, University of Bristol, Cantock’s Close, Bristol, UK; 2 BrisSynBio, University of Bristol, Life Sciences Building, Tyndall Avenue, Bristol, UK; 3 Department of Biochemistry, University of Bayreuth, Bayreuth, Germany; 4 School of Biochemistry, University of Bristol, University Walk, Bristol, UK

**Keywords:** Structural biology, Protein design, Protein design

## Abstract

De novo protein design is advancing rapidly. However, most designs are for single states. Here we report a de novo designed peptide that forms multiple α-helical-bundle states that are accessible and interconvertible under the same conditions. Usually in such designs amphipathic α helices associate to form compact structures with consolidated hydrophobic cores. However, recent rational and computational designs have delivered open α-helical barrels with functionalisable cavities. By placing glycine judiciously in the helical interfaces of an α-helical barrel, we obtain both open and compact states in a single protein crystal. Molecular dynamics simulations indicate a free-energy landscape with multiple and interconverting states. Together, these findings suggest a frustrated system in which steric interactions that maintain the open barrel and the hydrophobic effect that drives complete collapse are traded-off. Indeed, addition of a hydrophobic co-solvent that can bind within the barrel affects the switch between the states both in silico and experimentally.

## Abstract (web summary)

So far most of the de novo designed proteins are for single states only. Here, the authors present the de novo design and crystal structure determination of a coiled-coil peptide that assembles into multiple, distinct conformational states under the same conditions and further characterise its properties with biophysical experiments, NMR and MD simulations.

## Introduction

Given current abilities to generate proteins from scratch, both rationally and computationally^1^, there is no doubt that de novo protein design has come of age^2^. However, with some exceptions, the extent to which synthetic proteins can be endowed with the range of functionalities observed in natural proteins is less certain^3–5^. Advances are being made to introduce binding and catalytic functions into de novo proteins^1,6^, and synthetic proteins are being used in cells to interface and augment natural biological functions^7,8^. However, most attention has focused on achieving de novo proteins that access a single state, and then confirming this to high resolution by X-ray crystallography or nuclear magnetic resonance (NMR) spectroscopy. Moreover, many of the de novo designs made so far are hyper-thermally stable^9,10^. By contrast, most natural proteins are marginally stable. Recently, natural proteins have been engineered to introduce new, or to utilise inherent dynamics^11–15^. Such properties are critical for natural functions^16^, which includes the following: protein turnover and homoeostasis; conformational changes and allosteric effects involved in ligand binding, catalysis and release; and signal transduction. Ultimately, such protein functions afford biological systems exquisite control over their use of resources and responses to change. If these properties could be emulated in de novo proteins, then protein design would have truly come of age, and it will likely develop into a powerful tool with applications in biotechnology, synthetic biology and medicine.

To date, most de novo designs have targeted α-helical structures and assemblies^1^. This is largely because of established sequence-to-structure relationships and well-developed computational methods for such structures compared with those for designing structures based on β strands and sheets^9,10,17,18^. The de novo design of α-helical coiled coils has been particularly successful^19^. However, coiled coils are usually considered rigid rod-like structures^20^ stabilised by intimate side-chain interactions^21^. Thus, at first sight, they do not appear to be good candidates for conformational switching. However, it is increasingly apparent that natural coiled coils are dynamic and impart new biological functions^22–26^. For example, in influenza hemagglutinin, an extreme spring-like conformational transformation centres on a coiled coil^27^. Nonetheless, the design of coiled coils—and de novo proteins generally—that access multiple states under the same conditions has so far been elusive^28–37^. The challenge is to poise the stabilities of multiple target states such that no one state is favoured over another. This requires a deep understanding of the target structures.

**Fig. 1:**
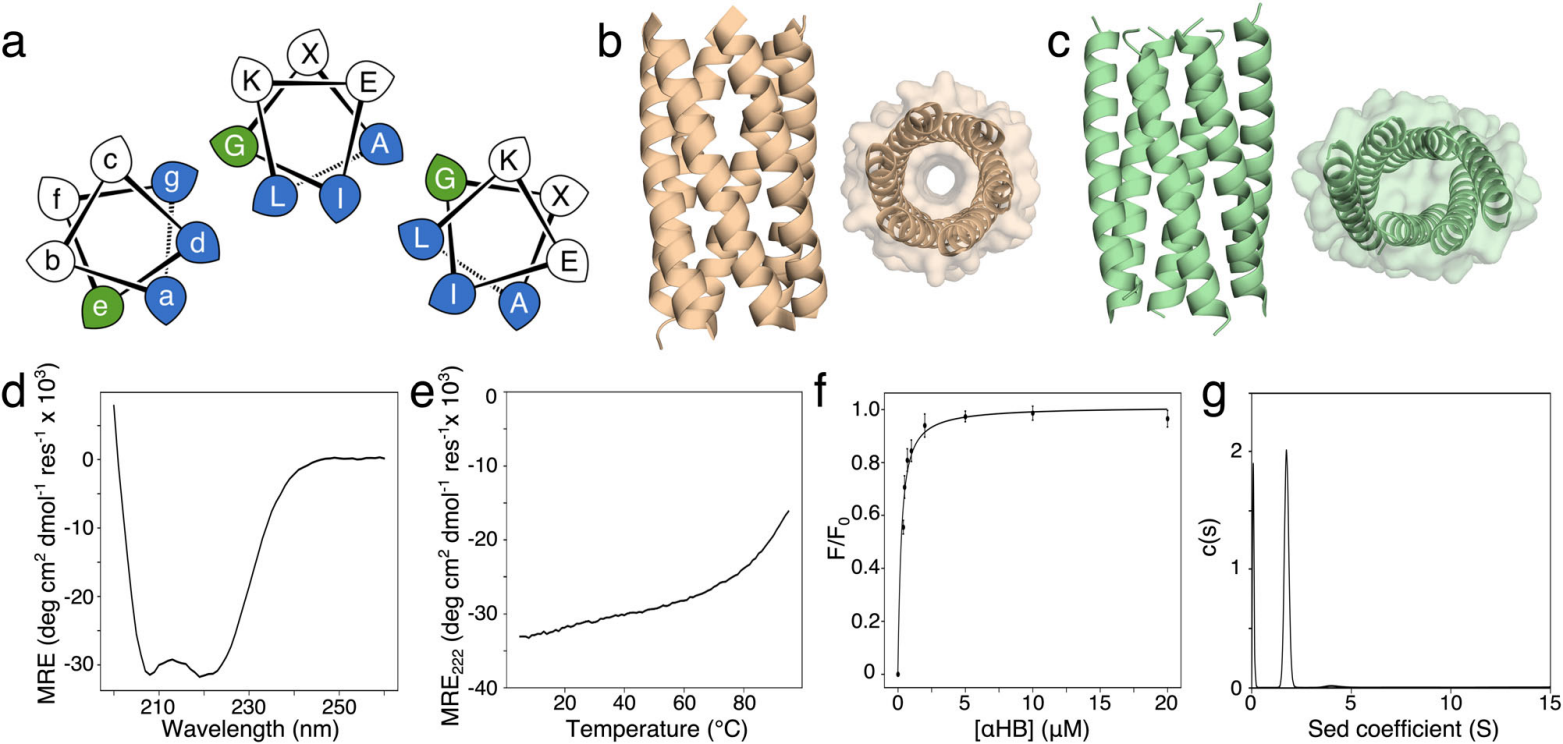
Design and characterisation of a dynamic coiled coil. **a** Helical wheel showing the gade interface (blue/green) of Type-2-coiled coils and the heptad repeat of CC-Type2-(L_a_I_d_G_e_)_4_. The Gly at the g position is highlighted (green). **b**, **c** Orthogonal views of X-ray crystal structures of an open hexameric α-helical barrel, CC-Type2-(S_g_L_a_I_d_)_4_ (straw; PDB code 4PN9), and a collapsed hexameric coiled coil, CC-Type2-(L_a_L_d_)_4_ (green; 6G6A). **d**, **e** CD spectrum (20 °C) and thermal denaturation profile (5–95 °C) for CC-Type2-(L_a_I_d_G_e_)_4_. MRE, mean residue ellipticity; MRE_222_, mean residue ellipticity at 222 nm. **f** Representative saturation binding curve for binding of diphenylhexatriene (DPH) to CC-Type2-(L_a_I_d_G_e_)_4_ monitored by fluorescence at 450 nm. Data presented as the mean ± SD; *K*_D_ = 230 ± 37 nM from *n* = 5 independent experiments. *F*/*F*_0_, fluorescence intensity ratio. **g** Sedimentation-velocity (AUC) of CC-Type2-(L_a_I_d_G_e_)_4_ with DPH, following the absorbance of DPH at 350 nm; molecular weight = 19,800 Da, corresponding to 6.2 × monomer mass. c(s), sedimentation coefficient distribution. Conditions: **d**, **e** 10 μM peptide; **f** 2.4–120 μM peptide, 0.1 μM DPH, 5% v/v DMSO; **g** 200 μM peptide, 5 μM DPH, 5% v/v DMSO. All experiments were performed in phosphate-buffered saline (PBS), pH 7.4.

In coiled coils, two or more α helices wrap around each other^25^. The helix–helix interactions are founded on knobs-into-holes (KIH) packing where ‘knob’ side chains from one helix dock into diamond-shaped ‘holes’ of side chains on neighbouring helices^21^. This is encoded by seven-residue sequence repeats called heptads and usually labelled abcdefg (Fig. 1a). Predominantly, residues at a and d are hydrophobic and drive the assembly of multiple amphipathic α helices. The majority of natural and designed coiled coils are dimers, trimers, or tetramers^38^. However, expanding the hydrophobic face to include the e and g sites leads to assemblies of five or more helices with or without central channels (Fig. 1b, c). The assemblies with channels are α-helical barrels (αHBs)^39^. These barrels can be designed both computationally and rationally to vary the size, shape and chemistry of the lumen, and to add functionality to it^9,10,40–43^. As with the lower-order classical coiled coils^44^, sequence-to-structure relationships have been elucidated that maintain αHBs and avoid alternative collapsed states^42^.

Here we describe the successful de novo design and structural resolution of a coiled-coil peptide that assembles into multiple, distinct conformational states under the same conditions. These are observed in both the crystal state and solution phase. The peptide forms an open αHB and a compact α-helical sandwich. Molecular dynamics (MD) simulations and NMR experiments show that these states interconvert and that it is possible to direct the switching with an external stimulus.

## Results

### A de novo α-helical-bundle accesses two distinct states simultaneously

Previously, we have described a de novo canonical αHB with the heptad repeat LKEIAxA^10^. This is named systematically as CC-Type2-(L_a_I_d_)_4_. We reasoned that introducing glycine (Gly, G) into this sequence should increase structural dynamics and potentially access alternative states, as Gly has the most backbone freedom of any α-amino acid, and it allows close approach of α helices^45^. The challenge was to place Gly to cause a conformational transition without compromising the oligomeric state and minimising the loss in overall stability. To do this, we considered the a, d, e or g sites at the helical interfaces of αHBs (Fig. 1a); deeming that the peripheral b, c and f sites would only affect overall stability and not the conformational states accessed. We discarded the a and d sites because Gly appears to be underrepresented here in known coiled-coil structures^38^, presumably because aliphatic hydrophobic side chains are required here to drive and stabilise coiled-coil formation. In addition, we understand now that β-branched side chains are required at d to maintain open αHB structures^42^. We also discarded placing Gly at g because, alongside residues at a and d, different side chains at this site can affect the oligomeric states of αHBs^10^. Therefore, we introduced Gly at all of the e sites to give CC-Type2-(L_a_I_d_G_e_)_4_ (Fig. 1a). This was made as a synthetic peptide, purified by high-performance liquid chromatography (HPLC) and confirmed by MALDI-TOF mass spectrometry (Supplementary Fig. 1 and Supplementary Table 1).

First, we characterised CC-Type2-(L_a_I_d_G_e_)_4_ in solution (Fig. 1d–g, and Supplementary Fig. 2). Circular dichroism (CD) spectroscopy showed that the peptide was α-helical at low μM concentrations. The parent peptide is hyper-thermal stable^10^. CD spectra of CC-Type2-(L_a_I_d_G_e_)_4_ recorded at increasing temperatures showed the start of an unfolding transition consistent with a destabilising effect of Gly^46^. Analytical ultracentrifugation (AUC) sedimentation-velocity and sedimentation-equilibrium experiments indicated that the peptide formed discrete hexamers (Supplementary Fig. 2). Titrations with diphenylhexatriene (DPH), which can bind within the lumens of αHBs, and further AUC experiments in the presence of DPH showed that CC-Type2-(L_a_I_d_G_e_)_4_ bound DPH consistent with an open-barrel state^42,43^.

**Fig. 2:**
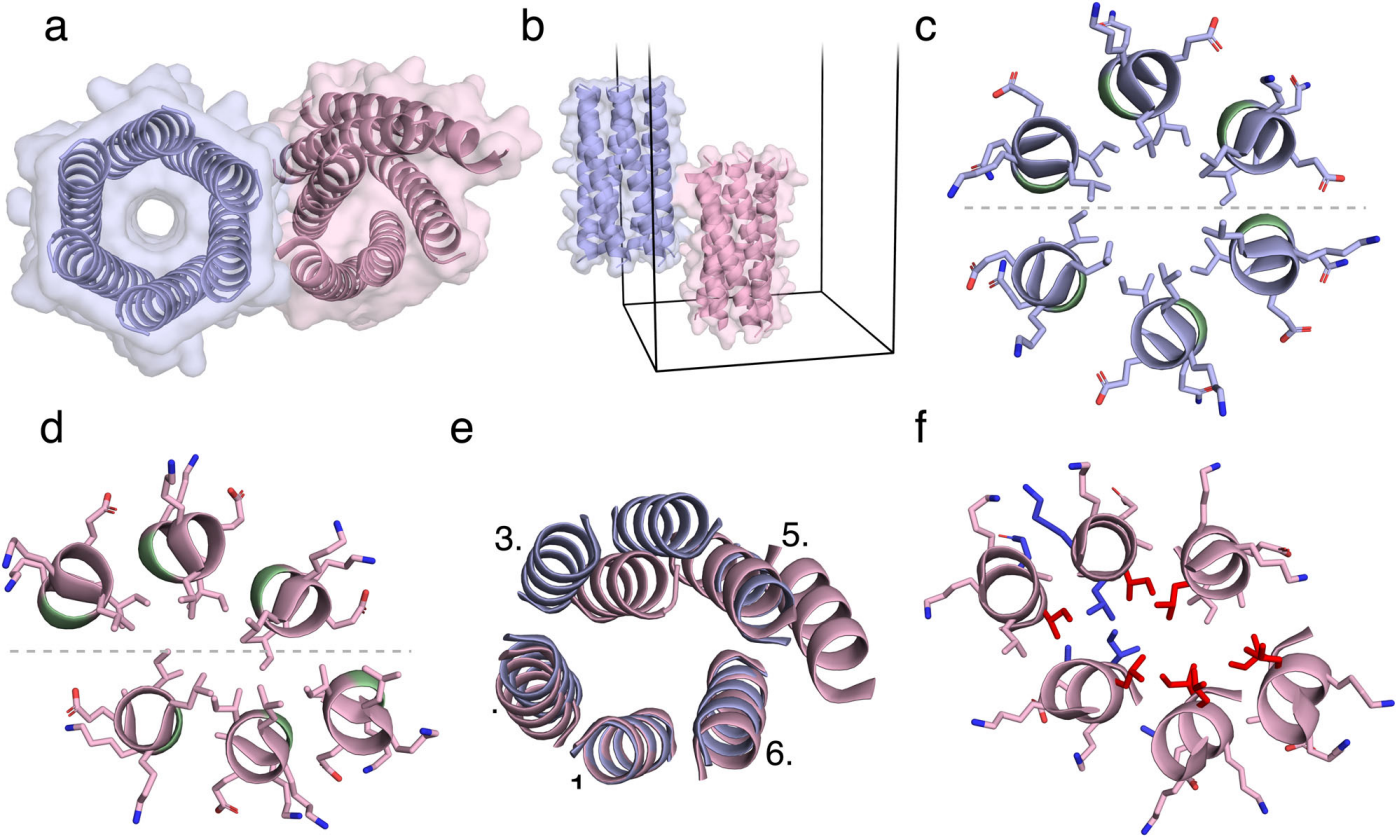
X-ray crystal structure of CC-Type2-(L_a_I_d_G_e_)_4_. **a**, **b** Orthogonal views of two states in the crystallographic unit cell (PDB entry 6ZT1). **c**, **d** Slices through the open (**c**) and collapsed (**d**) states showing side chains with the Gly residue at e sites highlighted green. The dashed lines highlight that both structures comprise similar three-helix sheets. **e** Overlay of the backbone structures of the two states. Two helical turns of each state for all chains are shown with the open C_6_-symmetric state in blue and the closed C_2_-symmetric state in red. The backbone root-mean-square deviation (RMSD) for the pair of helices labelled ‘1’ is 0.265 Å. **f** As in **d** with knobs-into-holes interactions highlighted with ‘cyclic’ knob residues coloured red and ‘non-cyclic’ and ‘inter-sheet’ knob residues coloured blue.

Next, we crystallised and solved an X-ray structure for a 4-bromophenylalanine variant of CC-Type2-(L_a_I_d_G_e_)_4_ (Fig. 2 and Supplementary Tables 1 and 2). To our surprise, the asymmetric unit contained two distinct and strikingly different α-helical hexamers (Fig. 2a, b): an open, C_6_-symmetric barrel; and a closed, C_2_-symmetric sandwich with a consolidated hydrophobic core. To a first approximation, both structures have two three-helix sheets with similar helix–helix interfaces and KIH interactions expected for Type-2 coiled coils (Fig. 2c, d). The main difference is that the two α-helical sheets of the open state are related cyclically to give the C_6_ symmetry; whereas, those of the closed state is sheared perpendicular to the central C_6_ axis, to give a C_2_-symmetric bundle. As a result, in the latter, the aliphatic residues form a compact hydrophobic core (Fig. 2d). Structural alignment revealed the two states to be related. Any helix from the open structure aligned to either of the central helices of the closed state (helices labelled ‘1’ in Fig. 2e). The three-helix substructures are closely similar with only small translations of their peripheral helices of 1.58 and 0.72 Å relative to each other (helices labelled ‘2’ and ‘6’, respectively, in Fig. 2e; Supplementary Table 3). However, relative to the open barrel, the remaining chains of the closed state are rearranged significantly with average translations in the paired helices ‘3’, ‘4’, and ‘5’ of 5.92, 6.98, and 6.05 Å, respectively (Fig. 2e). These larger structural changes are comparable to those found in natural allosteric proteins^16^.

We posit that the system is frustrated: in water, the hydrophobic effect drives towards collapsed helical bundles with consolidated, well-packed hydrophobic cores^19,47^. Opposing this, open α-helical barrels are maintained by steric interactions involving β-branched Ile residues at the d sites of the heptad repeats^42^. These form KIH interactions with residues Leu at a’, Ile at d’, and usually Ala at e’ of neighbouring helices^10,42^. We propose that the introduction of Gly at e’ in CC-Type2-(L_a_I_d_G_e_)_4_ perturbs this balance. The lack of side chain allows the helices to pack more closely together. This is apparent on closer examination of the structures: in the open state, the C_α_ atoms of the Ala-13 residue at g sites of neighbouring helices are spaced regularly at ≈9.3 Å; whereas, in the closed state the average distance is ≈8.8 Å. The freedom afforded by Gly at e also promotes breaking of the C_6_ symmetry. The helix–helix–helix angles are all ≈120˚ in the open state but vary between 90˚ and 150˚ in the close state. In turn, this allows the helices to pack less regularly in the closed state, leading to better optimised packing of aliphatic side chains. Indeed, analysis revealed ≈30% more KIH interactions in the closed state (Supplementary Fig. 3 and Supplementary Tables 4 & 5). In the open state, Leu and Ile residues at the a and d position engage in ‘cyclic’ KIH interactions^21^. The alternative collapsed state has significantly fewer of these classical interactions. However, this is compensated by new KIH interactions from the b, c, e and g positions and ‘inter-sheet’ interactions between Leu and Ile side chains (Fig. 2f). This trade-off between steric contributions favouring the open-barrel state and the hydrophobic effect driving towards collapsed bundles presents a possible mechanism for the system to switch between the two states.

### Mapping the free-energy landscape reveals multiple accessible states in aqueous solution

**Fig. 3:**
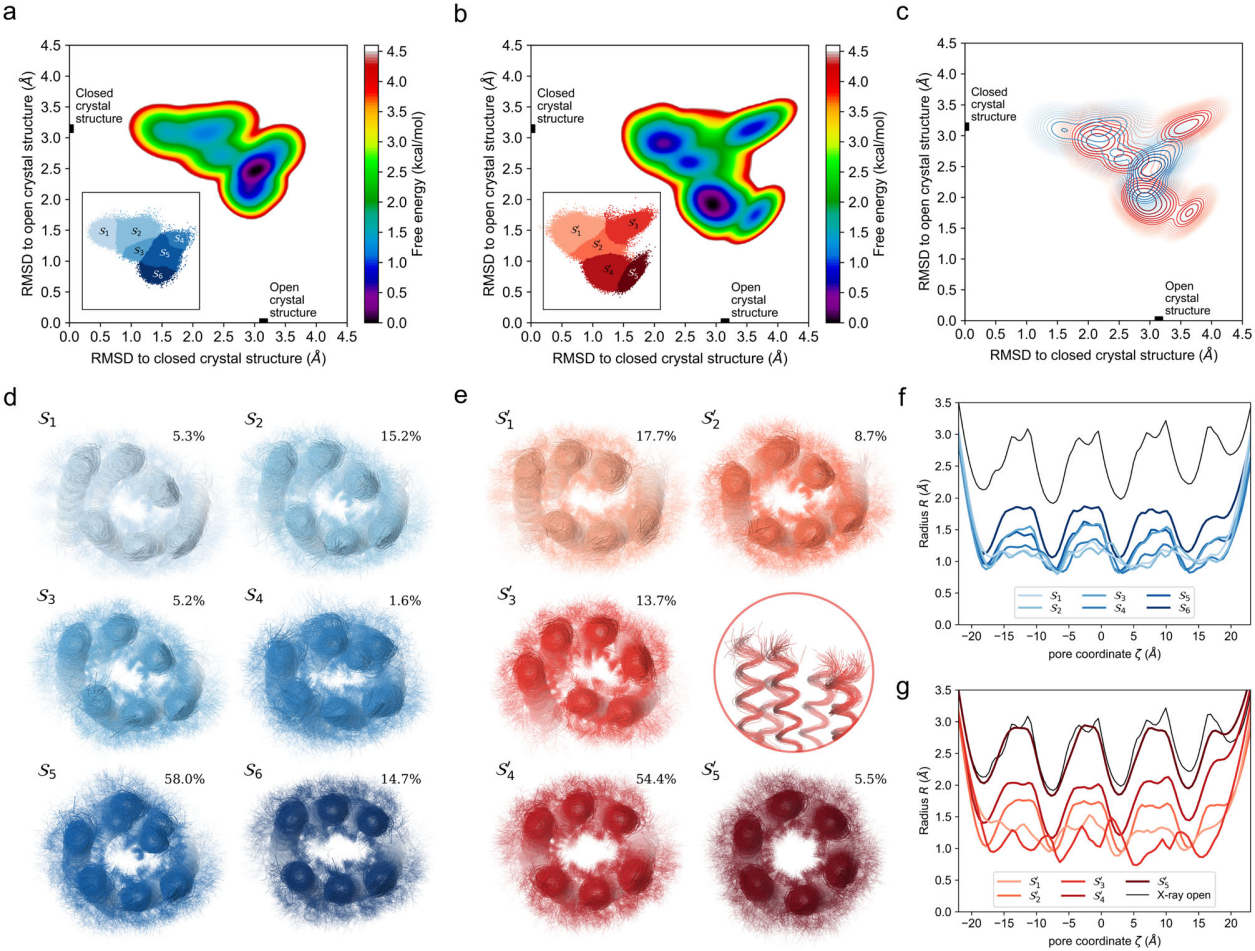
Molecular dynamics simulations of CC-Type2-(L_a_I_d_G_e_)_4_. **a**, **b** Conformational free-energy landscape calculated from multiple MD simulations. These were initiated from the open and closed crystal structures in the absence (**a**) and presence (**b**) of isopropanol, for a total simulation time of 54.7 μs and 83.5 μs, respectively. The root-mean-square deviations (RMSD) to the closed and open crystal structures (marked on the axes) provide conformational coordinates. Regions for the metastable states (S and S’) identified by clustering are shown as insets. **c** Superimposition of the free-energy minima in the absence (blue) and presence (red) of isopropanol. Each contour corresponds to 0.25 kcal/mol and their colour changes from dark to light as the energy increases. **d**, **e** Superimposed poses from each metastable state in the absence (**d**, S) and presence (**e**, S’) of isopropanol, with their relative population indicated in percentage. State S'_3_ is also shown from the side to highlight its slipped helices. **f**, **g** Mean channel radii of the metastable states in the absence (**f**) and presence (**g**) of isopropanol. The thin black lines are for the radius of the open channel in the crystal structure (the closed X-ray crystal structure is too collapsed to compute a channel radius).

To explore this as a possible mechanism, we conducted multiple all-atom MD simulations in water. These were initiated from both the open and closed crystal structures, to give a total simulation time of 54.7 µs. Previously, we have used this approach extensively to examine αHBs^42,43^. Indeed, work on CC-Type2-(S_g_L_a_I_d_)_4_, an open hexameric barrel in the crystal and solution phases, shows that it remains open and stable throughout simulations^43^. By contrast, the new MD simulations of CC-Type2-(L_a_I_d_G_e_)_4_ diverged from the symmetric open and closed states of the X-ray crystal structure to sample conformations between these (Fig. 3 and Supplementary Fig. 4). The resulting conformational free-energy landscape had a primary energy basin slightly closer to the open form, and a secondary shallower and broader region much closer to the closed form. Clustering the data using a Gaussian mixture model^48^ revealed six metastable core states (Fig. 3a & d and Supplementary Fig. 5a). These differed in slippage between the two three-helix sheets. At one extreme, state S_1_ deviated from the closed state X-ray crystal structure by a backbone root-mean-square deviation (RMSD) of just ≈1.5 Å, but this was only sparingly populated (≈5%). At the other extreme, state S_6_ was more open but only slightly more populated (≈15%). The dominant state at ≈60% occupancy, S_5_, lay approximately equidistant from the fully closed and open states of the X-ray crystal structure. Thus, overall, the states sample the region between the closed and open extremes of the experimental structure, though slightly skewed towards the former. The channel radii of the states were similar to each other, though narrowed compared with the fully open state from the X-ray crystal structure (Fig. 3f and Supplementary Fig. 6a). Moreover, even the most open of these states (S_6_) had a mean radius approximately half that of the experimental open state (Fig. 3f). Thus, the MD simulations indicate that the two crystal structure states are extrema in a dynamic equilibrium, as observed in natural allosteric proteins^49^. However, and importantly, the conformational space between them can be traversed, with the MD simulations suggesting that more-collapsed states are sampled in the majority.

In summary, the extensively sampled free-energy landscape supports the proposed mechanism in which the hydrophobic drive to a fully collapsed bundle is countered by side-chain steric interactions that specify an open barrel. This trade-off leaves multiple states poised, which allows alternate conformations of this de novo peptide assembly to be accessed under identical conditions and captured at high resolution (Fig. 2). Our next question was: could the position of the equilibrium be perturbed and controlled?

### Counteracting hydrophobic collapse enables control of the system

We targeted the hydrophobic effect as a means to control the equilibrium. Our hypothesis was that the system would switch towards the more-open forms upon addition of a more-hydrophobic solvent that could double as a small-molecule ligand and enter the hydrophobic lumen. We chose isopropanol (IPA) as it is part of the crystallisation conditions leading to X-ray crystal structures of large α-helical barrels with open hydrophobic lumens^42^, including CC-Type2-(L_a_I_d_G_e_)_4_.

First, further MD simulations were run in the presence of 25% v/v IPA. Multiple simulations were initiated from the open and closed crystal structures, plus others emanating from the main states using adaptive sampling, giving a total simulation time of 83.5 µs. The resulting conformational free-energy landscape (Fig. 3b) was shifted toward the more-open states compared with the simulations run in an aqueous buffer alone (Fig. 3a). In contrast to the simulations without IPA, there were multiple well-defined, lower-energy minima, separated by higher-energy barriers and a total of five metastable states (S’_1_ to S’_5_) were identified using the same clustering method (Fig. 3b, e and Supplementary Fig. 5b).

IPA appeared to alter the energy landscape moving it towards more-open structures rather than by simply changing the relative depths of energy basins observed in water alone (Fig. 3c): the metastable states S’_1_ and S’_2_ overlapped partly with S_2_ and S_3_, respectively; the most populated state, S’_4,_ overlapped with state S_6_; states similar to S_3_, S_4_ and S_5_ in water are only marginally sampled with IPA present; and states S’_3_ and S’_5_ were only sampled with IPA present. S’_5_ clearly identifies as the most open state with a backbone RMSD of ~1.5 Å from the open state in the X-ray crystal structure. However, and as with the closed state in aqueous solution (S_1_), S’_5_ only accounted ≈5% of the conformations sampled. Nonetheless, the states sampled with IPA were markedly more open than those observed in water alone (compare Figs. 3g, f). Indeed, the S’_5_ conformations were fully open (Fig. 3e and Supplementary Fig. 7) with mean channel radii close to that of the open X-ray crystal structure (Fig. 3g and Supplementary Fig. 6b), and their lumens were penetrated by IPA (Supplementary Fig. 8). The main state, S’_4_, was also slightly more open and accessible than the most open state in water, S_6_ (Supplementary Figs. 6 and 7).

Thus, simulations with IPA present suggest that hydrophobic co-solvents/small molecules counteract hydrophobic collapse and weaken van der Waals packing in the closed states of CC-Type2-(L_a_I_d_G_e_)_4_. We posit that this should emphasise the side-chain steric effects of Ile at d to push the equilibrium towards more-open states.

### A switch between species in solution

**Fig. 4:**
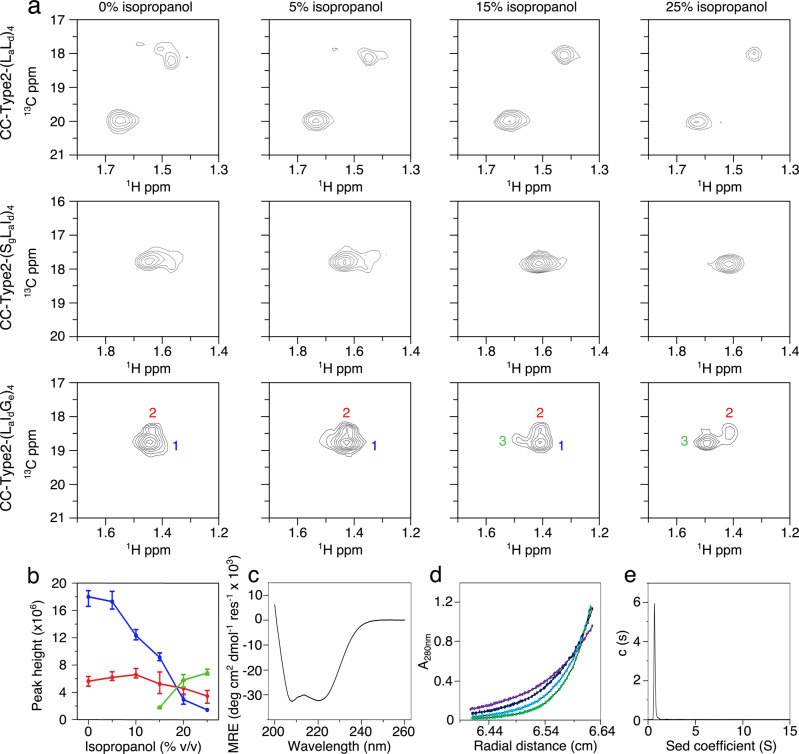
Solution-phase NMR studies of de novo coiled coils. **a** Regions of ^1^H-^13^C HSQC spectra showing ^13^C-Ala peaks of CC-Type2-(L_a_L_d_)_4_ (top), CC-Type2-(S_g_L_a_I_d_)_4_ (middle), and CC-Type2-(L_a_I_d_G_e_)_4_ (bottom) through isopropanol titrations. ppm, parts per million. **b** Peak heights of CC-Type2-(L_a_I_d_G_e_)_4_ from *n* = 3 independent experiments. These are for peaks labelled for CC-Type2-(L_a_I_d_G_e_)_4_ in **a** peak 1, blue; peak 2, red; peak 3, green. Data are presented as mean values ± range. **c** CD spectrum of CC-Type2-(L_a_I_d_G_e_)_4_ with 5% v/v isopropanol at 20 °C. MRE, mean residue ellipticity. **d** Sedimentation-equilibrium AUC data of CC-Type2-(L_a_I_d_G_e_)_4_ with 15% v/v isopropanol. Fitting to a single ideal species returned a weight of 18320 Da, ≈5.7 × monomer mass. Key: 30 krpm, purple; 35 krpm, navy; 40 krpm, light blue; 45 krpm, green. A, absorbance. **e** Sedimentation-velocity AUC data of CC-Type2-(L_a_I_d_G_e_)_4_ with 25% v/v isopropanol, which gave a weight of 17600 Da, ≈5.5 × monomer mass. c(s), sedimentation coefficient distribution. Peptide concentrations: **a**, **c** 300 μM; **d** 70 μM; and **e** 150 μM. All biophysical measurements were made in PBS pH 7.4.

To test if IPA did perturb the equilibrium ensemble experimentally, we turned to solution-phase NMR spectroscopy. To enable ^1^H-^13^C heteronuclear single-quantum coherence (HSQC) spectroscopy, we introduced ^13^C-methyl labels at Ala-13 in CC-Type2-(L_a_I_d_G_e_)_4_ and in two control peptides. Ala-13 is at a g site in the helical interfaces of CC-Type2-(L_a_I_d_G_e_)_4_. It samples different environments in the open and closed states (Fig. 2c, d), and, therefore, it is a potential reporter of conformational change. The first control was CC-Type2-(L_a_L_d_)_4_, a collapsed hexamer in solution and the crystal state (Fig. 1c). The ^1^H-^13^C HSQC spectrum had two resonances with peak heights in the ratio ≈2:1 (Fig. 4a and Supplementary Table 6). This is consistent with three environments of Ala-13 expected from the X-ray crystal structures of CC-Type2-(L_a_L_d_)_4_, two of which appear to have similar/identical chemical shifts (Supplementary Table 6)^42^. Moreover, aside from concomitant losses in intensity of both peaks, the spectra changed little through a titration with IPA, (Fig. 4a and Supplementary Table 6). This is consistent with this control peptide remaining as a collapsed, C_2_-symmetric bundle throughout the titration. The second control was CC-Type2-(S_g_L_a_I_d_)_4_, a canonical hexameric barrel^10^, with ^13^C-Ala at position 18, which is at an e position and in the helix-helix interface. In aqueous buffer and at low IPA, this gave a HSQC resonance broadened in the ^1^H dimension only, suggesting a small amount of conformational heterogeneity from very closely related states. However, this sharpened into a single stable peak at 10% IPA and above (Fig. 4a & Supplementary Table 6) consistent with the C_6_-symmetric barrel observed by X-ray crystallography (Fig. 1b)^10^.

By contrast, without IPA the labelled CC-Type2-(L_a_I_d_G_e_)_4_ sample gave two overlapping HSQC resonances, peaks ‘1’ and ‘2’, in the ratio ≈3:1 (Fig. 4a). The initial main peak ‘1’ was lost through the IPA titration and a new peak ‘3’ appeared, which became the main peak (Fig. 4a, b). We interpret these data as follows. Initially in water, multiple but similar species of lower symmetry are present resulting in peaks ‘1’ and ‘2’. Upon addition of IPA, Ala-13 increasingly encounters a different dominant environment, consistent with more-open, higher symmetry structures (Fig. 2c, Supplementary Table 6), which results in peak ‘3’. Increasing IPA binds to and stabilises the lumen of the open form to shift the equilibrium towards that state. This interpretation tallies with the in silico simulations that show IPA:αHB interactions promote open conformations similar to the αHB crystal form (Supplementary Fig. 7 and 9). In an attempt to assess and reconcile the data semi-quantitatively, we calculated the solvent accessible surface area (SASA) of Ala-13 Cβ of the states in the MD simulations (Supplementary Fig. 10). For the simulations in water, two environments—which we classify as buried and exposed—were observed with averaged occupancies of 74% and 26%, respectively. Interestingly, this is close to the 3:1 ratio of peak heights initially observed by NMR. Therefore, we contend that the equilibrium of states from MD simulations in water can explain the experimental NMR data. The calculations for the simulations in the presence of IPA revealed an increase in the occupancy of the exposed states to 36%, which correlates less well with the NMR data; though we note that we should be more circumspect in interpreting the SASA calculations in the presence of IPA (Supplementary Fig. 10).

To be sure that the peptide assemblies were robust to additions of IPA, we conducted further solution-phase biophysical studies using CD spectroscopy, AUC, and size exclusion chromatography. These confirmed that the peptide remained fully folded and hexameric in aqueous IPA (Fig. 4c–e and Supplementary Fig. 11).

## Discussion

We have presented the complete characterisation of a de novo designed peptide that assembles into distinct states under sets of identical conditions. Specifically, the α-helical peptide accesses distinct collapsed and open-barrel coiled-coil states simultaneously in a single crystal. Although there are only sub-Å differences in neighbouring-helix contacts between the two extreme states, the global effect is to move groups of helices by several Å. Detailed examination of the two extreme states from the X-ray crystal structure suggests an atomistic mechanism for switching between the states: from the basic tenet of protein folding, the collapsed state is favoured by the hydrophobic effect and the drive to form a consolidated hydrophobic core. However, for this class of coiled-coil assembly, collapse is opposed by β-branched residues, especially isoleucine, at the critical d position of the coiled-coil sequence repeat. This favours the open state by causing steric clashes in alternative collapsed forms^42^. In essence, the system is frustrated. The rational introduction of glycine residues into the sequence relaxes the restraint placed by isoleucine at d allowing closer helix–helix contacts to be made. In turn, these local changes allow the assembly to reconfigure into more-collapsed states. We show that this mechanism is likely to be in operation using extensive MD simulations. These define a free-energy landscape for the system and reveal multiple low-energy states that coexist in a dynamic equilibrium. Finally, we show that the equilibrium can be shifted in solution both in silico and experimentally by adding a hydrophobic co-solvent that can bind within the lumen of the open state.

The de novo design of dynamic multi-state proteins has been a long-standing challenge in protein design. Others have designed simple coiled-coil-based switches triggered by changes in pH^30,32,35,36^ and metal binding^28,29,31,33,37^. However, none of these has high-resolution data of both states under the same conditions. Moreover, the transduction of sub-Å inter-helix changes to larger global changes that we report is how cooperativity is manifest in classical natural systems such as haemoglobin^16^. Our findings should help pave the way to designing allosteric synthetic systems that emulate these natural proteins. In turn, this could allow responsive binding, catalysis and sensing to be built into de novo protein frameworks.

## Methods

### General

Fmoc-amino acids, dimethylformamide (DMF) and 6-chloro-1-hydroxybenzotriazole (Cl-HOBt) were purchased from Cambridge Reagents (Barton-upon-Humber, UK). N,N′-Diisopropylcarbodiimide was purchased from Carbosynth (Newbury, UK). All other reagents were purchased from Merck (Darmstadt, Germany).

Unless stated otherwise, all experiments were conducted in phosphate-buffered saline (PBS; 8.2 mM sodium phosphate dibasic, 1.8 mM potassium phosphate dibasic, 137 mM sodium chloride, 2.4 mM potassium chloride, pH 7.4). Peptide concentration was determined at 280 nm using a ThermoScientific (Hemel Hemstead, UK) Nanodrop 2000 spectrometer (*ε*_280_ = 5690 cm^−1^).

### Solid-phase peptide synthesis

Peptides were synthesised by standard Fmoc-chemistry using a CEM (Buckingham, UK) Liberty Blue automated peptide synthesis apparatus with inline UV monitoring. Activation was achieved with DIC/Cl-HOBt and Fmoc deprotection with 20% v/v morpholine/DMF. All coupling and deprotection steps were performed at 75 °C. Peptides were synthesised as *C-*terminal amides on Rink amide MBHA solid-support and *N-*terminally acetylated using pyridine (0.3 mL) and acetic anhydride (0.25 mL) in DMF (10 mL) for 20 min at room temperature (rt). Subsequent cleavage from the solid support was achieved with trifluoroacetic acid (TFA):triisopropylsilane:H_2_O in a 95:2.5:2.5 ratio for 3 h at room temperature. The cleavage solution was evaporated under nitrogen (to ~5 mL) before the peptide was precipitated in cold diethyl ether and recovered via centrifugation (10 min, 1620 × *g*). Crude peptide was dissolved in 50:50 v/v acetonitrile:H_2_O before lyophilisation to yield a white or off-white solid.

### Peptide purification and characterisation

Peptides were purified by reverse phase HPLC (Jasco; Great Dunnow, UK) using a Phenomenex (Macclesfield, UK) Luna C18 stationary phase column (150 × 10 mm, 5 μM particle size, 100 Å pore size). A 40–100% gradient of acetonitrile in water (with 0.1% v/v TFA) over 30 min at 50 °C was used to separate the peptide. Pure peptide was identified via analytical HPLC and MADLI-TOF.

Analytical HPLC was performed using a Phenomenex Kinetix C18 stationary phase column (100 × 4.6 mm, 5 μM particle size, 100 Å pore size). A 40–100% gradient of acetonitrile in water (with 0.1% v/v TFA) over 15 min at 50 °C was used to analyse peptide purity.

MALDI-TOF was performed on a Bruker (Coventry, UK) Ultraflex MALDI-TOF mass spectrometer operating in positive-ion reflector mode. Peptides were spotted on a ground steel plate using dihydroxybenzoic acid or α-cyano-4-hydroxycinnamic acid as the matrix. Masses quoted are for the monoisotopic mass of the singly protonated species, [M + H]^+^.

### CD spectroscopy

CD spectroscopy was performed on a Jasco J-810 or J-815 spectropolarimeter fitted with a Peltier temperature controller. Data were collected in a 1 or 5 mm quartz cuvette between 190 and 260 nm using a scan rate of 100 nm min^−1^, a 1 nm interval and bandwidth and a response time of 1 s. Standard CD spectra were acquired at 10 μM peptide concentration at 20 °C. Thermal denaturation spectra were collected at 222 nm using the settings and peptide concentration as above. CD spectra in the presence of IPA were acquired at 300 μM peptide concentration at 25 °C between 0 and 25% v/v IPA.

### Size-exclusion chromatography

Size-exclusion chromatography was performed using a Superdex75 10/300 GL column with 25% v/v IPA in PBS. Samples were loaded at 150 μM and monitored at 220 nm using a JASCO UV-vis detector.

### Analytical ultracentrifugation

AUC was performed on a Beckman Optima X-LA or X-LI analytical ultracentrifuge with an An-50-Ti or An-60-Ti rotor (Beckman-Coulter; Indianapolis, US). Buffer densities and viscosities and peptide partial specific volumes (v̅) were calculated using SEDNTERP (http://rasmb.org/sednterp/). The density and viscosity of 5% v/v dimethyl sulfoxide (DMSO) and 5–25% v/v IPA in H_2_O was taken from the literature^50^.

Standard AUC sedimentation-velocity (SV) experiments were performed at 150 μM peptide concentration at 20 °C. Data were collected in two-channel epon or aluminium centrepieces with quartz windows at 50 krpm at 5-minute intervals for a total of 120 scans. The baseline, meniscus, frictional coefficient (*f*/*f*_0_) and systematic time-invariant and radial-invariant noise were fitted using SEDFIT^2^ at a 95% confidence level. Residuals are shown as a bitmap where greyscale shade indicates the difference between the model and raw data over the radial range of the fit (residuals < −0.05 black, >0.05 white), with scans ordered vertically from the top of the image.

AUC SV experiments in the presence of DPH were carried out at 200 μM peptide concentration, 5 μM DPH, 5% v/v DMSO in PBS at 25 °C. Experimental conditions and data processing were carried out as above. AUC sedimentation-equilibrium experiments were performed at 70 μM peptide concentration at 20 °C. Data were collected in six-channel epon centrepieces with quartz windows between 15 and 30 krpm with a minimum of three speeds sampled after equilibration for 8 h. Data were fitted to a single species model using SEDPHAT^51^. Monte Carlo analysis was performed on each fit to give 95% confidence limits.

### Ligand binding

Ligand-binding experiments were performed on an epMotion 5070 liquid handler (Eppendorf; Hamburg, Germany). The total concentration of ligand was kept constant (0.1 μM) and the concentration of αHB varied from 0–50 μM. All assays had 5% v/v DMSO added to solubilise the ligand. Data were collected after 2 h equilibration on a BMG Labtech (Aylesbury, UK) Clariostar plate reader using an excitation wavelength of 350 nm and emission monitored between 400–600 nm. Binding constants were extracted by fitting Eq. (1)^52^ to the data in SigmaPlot 13.0.

1 $$y = B_{\mathrm{max}}\frac{{\left( {c + x + K_D} \right) + \sqrt {\left( {c + x + K_D} \right)^2\, -\, 4cx} }}{{2c}}$$

Where *c* is the total concentration of the constant component (e.g., DPH), *x* is the concentration of variable component (e.g., αHB), *B*_max_ is the fluorescence signal when all of the constant component is bound, and *y* is the fraction of bound component being monitored via fluorescence signal and *K*_*D*_ is the dissociation constant.

### X-ray crystal structure determination

Freeze-dried peptide was dissolved in deionized water to an approximate concentration of 10 mg ml^−1^ for vapour-diffusion crystallisation trials using standard commercial screens (JCSG, Structure Screen 1+2, ProPlex, PACT Premier and Morpheus^TM^) at 19 °C with 0.3 μL of the peptide solution equilibrated with 0.3 μL of the screen solution. To aid with cryoprotection, crystals were soaked in their respective reservoir solutions containing 25% glycerol prior to data collection. Diffracting crystals were obtained in 0.05 M Sodium HEPES, 5% w/v PEG 4000 and 5% v/v 2-propanol at a pH of 7 (values based on 1:1 dilution of ProPlex B6 crystallisation condition with peptide solution). X-ray diffraction data were collected at the Diamond Light Source (Didcot, UK) at a wavelength of 0.919 Å on beamline I24 for CC-Type2-(L_a_I_d_G_e_)_4_-W19BrPhe. Data were processed using the Xia2 3dii autoprocessing pipeline^53^. The 3dii pipeline uses XDS for point-group selection and integration and XSCALE for scaling and merging of diffraction data^54^. Experimental phasing and structure building were achieved using the Big EP automated pipeline^55^, which utilises autoSHARP^56^, Phenix AutoSol/AutoBuild^57^ and Crank2^58^. The data set was phased by SAD phasing of bromine atoms from 4-bromophenylalanine residues. The final structure was obtained after iterative rounds of model building with COOT^59^ and refinement with Refmac 5^60^. Solvent-exposed side-chain atoms lacking map density were modelled at zero occupancy, which was identified as unobserved and labelled in the coordinate file under REMARK 480. The coordinate file and structure factor file were deposited in the Protein Data Bank (PDB ID: 6ZT1). All images of the crystal structure were generated with PyMOL (www.pymol.org).

### MD system preparation

Starting from the open and closed form from the crystal structure of CC-Type2-(L_a_I_d_G_e_)_4_ (PDB ID: 6ZT1), bromophenylalanine was mutated to phenylalanine in Pymol and missing terminal residues were modelled in using the automodel routine of Modeller^61^ and with other residues kept fixed. Crystallographic water molecules were retained and Maestro’s Protein Preparation Wizard (Schrödinger Release 2017-4: Maestro, Schrödinger, LLC, New York, NY, 2020) was used to add hydrogens, choose between possible flipped side-chain conformations, H-bond network optimisations, and capping of the terminal residues. The SOLVATE programme (http://www.mpibpc.mpg.de/grubmueller/solvate) was used to create a solvation shell of TIP3P^62^ water molecules of at least 5 Å around the protein using eight Gaussians.

From the resulting pre-solvated structures, two sets of systems were prepared: one in the absence and the other in the presence of IPA, while the peptides were modelled with the Amber ff14SB force field^63^. To model IPA, the force field parameters were those developed by Jorgensen for the OPLS force field^64^ as they were identified as the best ones for running simulations of water:IPA in MixMD^65^ (the Carlson group kindly shared the Amber version of those parameters along with their scripts).

For the simulations in water only, a truncated octahedron cell of TIP3P water was created with tleap, part of the AmberTools17 modelling suite^66^, with padding to 10 Å around the previously generated solvation shell and the closeness to 0.75 Å. Na^+^ and Cl^−^ ions were added randomly to the solvent cell to neutralise the protein charge and model a concentration of 0.1 M NaCl.

For the simulations in the presence of IPA, a 25%/75% IPA/Water mixture was used. A truncated octahedron cell of IPA was created around the initial solvation shell obtained with SOLVATE with a padding of 6 Å and closeness of 0.5 Å. The optimal size of the truncated octahedron cell of water to be added on top of this was then determined via a binary search. As for the water only systems, Na^+^ and Cl^−^ ions were added to a concentration of 0.1 M NaCl.

For both systems, hydrogen mass repartitioning^67^ was used to allow increasing the timestep of the simulations to 4 fs.

### MD simulations

For each solvent system (i.e., water only or water:IPA), simulations were initiated from each crystallographic state (i.e., open or closed). For each simulation, prior to production MD, the systems were carefully minimised, heated and equilibrated independently. Minimisation and heating were performed using the parallel CPU version of pmemd (part of Amber) and a timestep of 2 fs, whereas equilibration and production MD were run on single GPUs using the cuda version of pmemd^68^ and a timestep of 4 fs. Simulations were run using periodic boundary conditions, SHAKE to constraint bonds involving a hydrogen atom and Particle Mesh Ewald summation method with a cutoff of 10 Å for long-range electrostatics. Simulations were run indifferently with the Amber16 and Amber18 versions of pmemd. The difference between the two versions for a given starting structure and seed number were found to be minimal and in no way detrimental to the present study for which we sought to maximise the sampling of the conformational space.

The minimisation procedure was carried out by first restraining the entire protein to relax solvent and ions, then only backbone atoms were restrained to relax the side chains, then only Cα were restrained, and finally the entire system was minimised without any restraints. For each of those minimisations, the steepest descent algorithm was used for the first 1000 steps followed by 10,000 steps using the conjugate gradient algorithm. After minimisation, the system was heated by increasing the temperature linearly to 293.15 K over 100 ps, using Langevin dynamics with a collision frequency of 5 ps^−1^, restraining backbone atoms with a 5 kcal mol^−1^ Å^−2^ force constant. Heating was followed by NPT equilibration using a Monte Carlo barostat with a pressure relaxation time of 1 ps to maintain the pressure at 1.01325 bar, and decreasing the restraints on backbone atoms from 5 to 1 kcal mol^−1^ Å^−2^ force constant over 1.5 ns by steps of 500 ps, then switching the restraint on Cα only form 1 to 0.25 kcal mol^−1^ Å^−2^ force constant over 1.5 ns by steps of 500 ps. Finally, an equilibration of 10 ns without any restraints was carried out.

Production MD was performed in the NPT ensemble using the same parameters as for the equilibration. In the absence of IPA, 10 simulations were initiated from the open form and 10 from the closed form, with the simulations initially ran for ~2.5 μs and some were continued further if several transitions between distinct regions of the energy landscape were sampled, leading to a total of 54.7 μs simulated. In the presence of IPA, 10 simulations were initiated from the closed form and 10 from the open form, initially for 2.5 μs and running for longer the simulations where several transitions were observed. Construction of an initial free-energy landscape revealed that the sampling was not yet sufficient, especially for the states sampled primarily when using the open form as starting structure. To alleviate this, five additional 2.5 μs simulations were initiated from the open form and then, after identifying the main states of the system, a total of 24 additional simulations were initiated from each metastable state identified (five per state, four for the most populated state) and were run for 500 ns. This led to a total of 83.5 μs simulated in the presence of IPA.

### MD analysis

We constructed the free-energy landscape of the system, both in the presence and absence of IPA using the backbone RMSD to the open and closed crystal structures as conformational coordinates (or features) to describe the transition between open and closed forms.

The closed crystal structure has a specific topology for its chain names, with the dimer interface separating the trimer composed of chains named B, C and D from the trimer composed of chains named E, F and A. However, simulations initiated from the open crystal structure can vary in chain name topology (e.g., the dimer interface can be between trimers A, B, C and D, E, F). Because the frames of a trajectory need to be aligned to a reference structure to calculate the RMSD, alignment of the simulations initiated from the open crystal structure to the closed crystal structure led to large RMSD values simply because the topology of the chain names was different. To alleviate this artefact, we generated the six possible chain name topologies for the closed form simply obtained by rotating the name of the chains (the possible chain name topologies for the dimer of trimers are [(A B C) (D E F)], [(B C D) (E F A)], [(C D E) (F A B)], [(D E F) (A B C)], [(E F A) (B C D)], [(F A B) (C D E)], and calculated the RMSD to each of those versions of the same closed form and retained the lowest RMSD values. RMSD calculations were performed with PYTRAJ^69^.

The free-energy landscape along the RMSD coordinates was then estimated using the InfleCS method that uses Gaussian mixture models (GMM)^48^. The free-energy landscapes were estimated with GMM estimators constructed with 151 grids using 20 iterations. The optimum number of components for the GMM was found to be 10 in the absence of IPA and 13 in the presence of IPA. The initial clustering was performed using 1/5th of the frames, and the full dataset was then assigned to the different clusters.

In order to check the convergence of the simulations, the analysis was repeated by randomly selecting only 1/50th of the total number of frames. The results for the full and reduced dataset were comparable (Supplementary Fig. 12), indicating that the MD sampling was sufficient to describe this region of the free-energy landscape.

In order to extract representative conformations for each core state, a second, ‘crisp’ clustering was performed with the ‘assign_transition_points=False option so that only the low-energy conformations were taken into account, alleviating the need to weight the conformations according to the stationary distribution (Supplementary Fig. 13). From each core state, 1000 conformations were sampled from the crisp clustering and were used to calculate the average channel radius and standard deviation using HOLE^70^. IPA density maps were computed using CPPTRAJ^69^ and rendered with VMD^71^.

The SASA for Ala-13 Cβ was calculated with CPPTRAJ^69^ for each chain for the 1000 conformations sampled for each core states. Two environments were considered for the analysis: buried (SASA < 10 Å^2^) or exposed (SASA > 10 Å^2^), The proportion of exposed vs. buried observed in solution was obtained by weighting the proportion of exposed vs. buried Ala-13 Cβ for each state with the relative population of each state.

### NMR spectroscopy

All NMR spectroscopy experiments were performed at 298 K on a Bruker Avance III HD 700 MHz spectrometer equipped with a 1.7 mm inverse triple-resonance micro-cryocoil probe. NMR samples were prepared by dissolving the appropriate ^13^C-labelled freeze–dried peptide in PBS at pH 7.4 in 100% D_2_O to a final concentration of 300 μM. 2-propanol-d_8_ was added to a final concentration of 25% (v/v) in 5% increments. Band-selective ^1^H-^13^C HSQC experiments centred at 17.6 ppm in f1 were acquired using the standard Bruker pulse sequence shsqcetgpsisp2.2 with a relaxation delay of 1 sec and spectra widths of 10504.2 Hz in f1 and 2875.2 Hz in f2 with 50% non-uniform sampling. Spectra were referenced against trimethylsilylpropanoic acid. NMR data were processed with NMRPipe^72^ and qMDD^73,74^. Analysis was carried out in CCPNMR Analysis version 2.4.2^75^.

### Reporting summary

Further information on experimental design is available in the Nature Research Reporting Summary linked to this paper.

## Supplementary information

Supplementary Information

Peer Review File

Reporting Summary

## Data Availability

The coordinate and structure factor files for CC-Type2-(L_a_I_d_G_e_)_4_ have been deposited in the Protein Data Bank with accession code 6ZT1. Script and compressed data files for the MD simulations are available at https://github.com/eric-jm-lang/MD-switch-paper. All other data presented in the study are available from the corresponding author on request. Source data are provided with this paper.

## Source data

Source Data

## References

[1] Korendovych IV, DeGrado WF (2020). De novo protein design, a retrospective. Q. Rev. Biophys..

[2] Huang PS, Boyken SE, Baker D (2016). The coming of age of de novo protein design. Nature.

[3] Ambroggio XI, Kuhlman B (2006). Design of protein conformational switches. Curr. Opin. Struc. Biol..

[4] Davey JA, Chica RA (2012). Multistate approaches in computational protein design. Protein Sci..

[5] Dawson WM, Rhys GG, Woolfson DN (2019). Towards functional de novo designed proteins. Curr. Opin. Chem. Biol..

[6] Studer S (2018). Evolution of a highly active and enantiospecific metalloenzyme from short peptides. Science.

[7] Smith AJ, Thomas F, Shoemark D, Woolfson DN, Savery NJ (2019). Guiding biomolecular interactions in cells using de novo protein-protein interfaces. ACS Synth. Biol..

[8] Lebar T, Lainscek D, Merljak E, Aupic J, Jerala R (2020). A tunable orthogonal coiled-coil interaction toolbox for engineering mammalian cells. Nat. Chem. Biol..

[9] Huang PS (2014). High thermodynamic stability of parametrically designed helical bundles. Science.

[10] Thomson AR (2014). Computational design of water-soluble alpha-helical barrels. Science.

[11] Suzuki Y (2016). Self-assembly of coherently dynamic, auxetic, two-dimensional protein crystals. Nature.

[12] Davey JA, Damry AM, Goto NK, Chica RA (2017). Rational design of proteins that exchange on functional timescales. Nat. Chem. Biol..

[13] Alberstein R, Suzuki Y, Paesani F, Tezcan FA (2018). Engineering the entropy-driven free-energy landscape of a dynamic nanoporous protein assembly. Nat. Chem..

[14] Chen KM, Keri D, Barth P (2020). Computational design of G protein-coupled receptor allosteric signal transductions. Nat. Chem. Biol..

[15] Crean RM, Gardner JM, Kamerlin SCL (2020). Harnessing conformational plasticity to generate designer enzymes. J. Am. Chem. Soc..

[16] Kuriyan, J., Konforti, B. & Wemmer, D. *The Molecules of Life**:**First Edition*. (Garland Science, Taylor & Francis Group, 2012).

[17] Grigoryan G, DeGrado WF (2011). Probing designability via a generalized model of helical bundle geometry. J. Mol. Biol..

[18] Wood CW, Woolfson DN (2018). CCBuilder 2.0: powerful and accessible coiled-coil modeling. Protein Sci..

[19] Woolfson DN (2017). Coiled-coil design: updated and upgraded. Subcell. Biochem..

[20] Rose A, Meier I (2004). Scaffolds, levers, rods and springs: diverse cellular functions of long coiled-coil proteins. Cell Mol. Life. Sci..

[21] Walshaw J, Woolfson DN (2001). SOCKET: a program for identifying and analysing coiled-coil motifs within protein structures. J. Mol. Biol..

[22] Hulko M (2006). The HAMP domain structure implies helix rotation in transmembrane signaling. Cell.

[23] Kon T (2009). Helix sliding in the stalk coiled coil of dynein couples ATPase and microtubule binding. Nat. Struct. Mol. Biol..

[24] Stewart CM (2016). Coiled-coil destabilizing residues in the group A Streptococcus M1 protein are required for functional interaction. Proc. Natl Acad. Sci. USA.

[25] Lupas AN, Bassler J (2017). Coiled coils - a model system for the 21st century. Trends Biochem. Sci..

[26] Snoberger A, Brettrager EJ, Smith DM (2018). Conformational switching in the coiled-coil domains of a proteasomal ATPase regulates substrate processing. Nat. Commun..

[27] Benton DJ, Gamblin SJ, Rosenthal PB, Skehel JJ (2020). Structural transitions in influenza haemagglutinin at membrane fusion pH. Nature.

[28] Cerasoli E, Sharpe BK, Woolfson DN (2005). ZiCo: A peptide designed to switch folded state upon binding zinc. J. Am. Chem. Soc..

[29] Ambroggio XI, Kuhlman B (2006). Computational design of a single amino acid sequence that can switch between two distinct protein folds. J. Am. Chem. Soc..

[30] Zimenkov Y (2006). Rational design of a reversible pH-responsive switch for peptide self-assembly. J. Am. Chem. Soc..

[31] Dublin SN, Conticello VP (2008). Design of a selective metal ion switch for self-assembly of peptide-based fibrils. J. Am. Chem. Soc..

[32] Lizatovic R (2016). A de novo designed coiled-coil peptide with a reversible pH-induced oligomerization switch. Structure.

[33] Aupic J, Lapenta F, Jerala R (2018). SwitCCh: metal-site design for controlling the assembly of a coiled-coil homodimer. ChemBioChem.

[34] Mueller C, Grossmann TN (2018). Coiled-coil peptide beacon: a tunable conformational switch for protein detection. Angew. Chem. Int. Ed..

[35] Boyken SE (2019). De novo design of tunable, pH-driven conformational changes. Science.

[36] Rhys GG (2019). Navigating the structural landscape of de novo alpha-helical bundles. J. Am. Chem. Soc..

[37] Wei KY (2020). Computational design of closely related proteins that adopt two well-defined but structurally divergent folds. Proc. Natl. Acad. Sci. USA.

[38] Testa OD, Moutevelis E, Woolfson DN (2009). CC plus: a relational database of coiled-coil structures. Nucl. Acids Res..

[39] Liu J (2006). A seven-helix coiled coil. Proc. Natl Acad. Sci. USA.

[40] Burgess NC (2015). Modular design of self-assembling peptide-based nanotubes. J. Am. Chem. Soc..

[41] Burton AJ, Thomson AR, Dawson WM, Brady RL, Woolfson DN (2016). Installing hydrolytic activity into a completely de novo protein framework. Nat. Chem..

[42] Rhys GG (2018). Maintaining and breaking symmetry in homomeric coiled-coil assemblies. Nat. Commun..

[43] Thomas F (2018). De novo-designed alpha-helical barrels as receptors for Small Molecules. ACS Synth. Biol..

[44] Harbury PB, Zhang T, Kim PS, Alber T (1993). A switch between 2-stranded, 3-stranded and 4-stranded coiled coils in Gcn4 leucine-zipper mutants. Science.

[45] Dong H, Sharma M, Zhou H-X, Cross TA (2012). Glycines: role in α-helical membrane protein structures and a potential indicator of native conformation. Biochemistry.

[46] Chakrabartty A, Schellman JA, Baldwin RL (1991). Large differences in the helix propensities of alanine and glycine. Nature.

[47] Bryson JW (1995). Protein design: a hierarchic approach. Science.

[48] Westerlund AM, Delemotte L (2019). InfleCS: clustering free energy landscapes with Gaussian mixtures. J. Chem. Theory Comput..

[49] Kern D, Zuiderweg ER (2003). The role of dynamics in allosteric regulation. Curr. Opin. Struct. Biol..

[50] LeBel RG, Goring DAI (1962). Density, viscosity, refractive index, and hygroscopicity of mixtures of water and dimethyl sulfoxide. J. Chem. Eng. Data.

[51] Schuck P (2003). On the analysis of protein self-association by sedimentation velocity analytical ultracentrifugation. Anal. Biochem..

[52] Kovacs E, Tóth J, Vértessy BG, Liliom K (2010). Dissociation of calmodulin-target peptide complexes by the lipid mediator sphingosylphosphorylcholine: implications in calcium signaling. J. Biol. Chem..

[53] Winter G (2010). xia2: an expert system for macromolecular crystallography data reduction. J. Appl. Crystallogr..

[54] Kabsch W (2010). XDS. Acta Crystallogr. D..

[55] Sikharulidze I, Winter G, Hall DR (2016). Big EP: automated structure solution pipeline deployment at diamond light source. Acta Crystallogr. A.

[56] Vonrhein, C., Blanc, E., Roversi, P. & Bricogne, G. in *Macromolecular Crystallography Protocols: Volume 2: Structure Determination* (ed Sylvie Doublié) 215–230 (Humana Press, 2007).

[57] Terwilliger TC (2009). Decision-making in structure solution using Bayesian estimates of map quality: the PHENIX AutoSol wizard. Acta Crystallogr. D..

[58] Skubák P, Pannu NS (2013). Automatic protein structure solution from weak X-ray data. Nat. Commun..

[59] Emsley P, Lohkamp B, Scott WG, Cowtan K (2010). Features and development of Coot. Acta Crystallogr. D..

[60] Murshudov GN (2011). REFMAC5 for the refinement of macromolecular crystal structures. Acta Crystallogr. D..

[61] Sali A, Blundell TL (1993). Comparative protein modelling by satisfaction of spatial restraints. J. Mol. Biol..

[62] Jorgensen WL, Chandrasekhar J, Madura JD, Impey RW, Klein ML (1983). Comparison of simple potential functions for simulating liquid water. J. Chem. Phys..

[63] Maier JA (2015). ff14SB: improving the accuracy of protein side chain and backbone parameters from ff99SB. J. Chem. Theory Comput..

[64] Jorgensen WL (1986). Optimized intermolecular potential functions for liquid alcohols. J. Phys. Chem..

[65] Lexa KW, Goh GB, Carlson HA (2014). Parameter choice matters: validating probe parameters for use in mixed-solvent simulations. J. Chem. Inf. Model..

[66] Case, D. A. et al. *AMBER 2017*, University of California (2017).

[67] Hopkins CW, Le Grand S, Walker RC, Roitberg AE (2015). Long-time-step molecular dynamics through hydrogen mass repartitioning. J. Chem. Theory Comput..

[68] Salomon-Ferrer R, Götz AW, Poole D, Le Grand S, Walker RC (2013). Routine microsecond molecular dynamics simulations with amber on gpus. 2. explicit solvent particle mesh Ewald. J. Chem. Theory Comput..

[69] Roe DR, Cheatham TE (2013). PTRAJ and CPPTRAJ: software for processing and analysis of molecular dynamics trajectory data. J. Chem. Theory Comput..

[70] Smart OS, Neduvelil JG, Wang X, Wallace BA, Sansom MSP (1996). HOLE: a program for the analysis of the pore dimensions of ion channel structural models. J. Mol. Graph..

[71] Humphrey W, Dalke A, Schulten K (1996). VMD: visual molecular dynamics. J. Mol. Graph..

[72] Delaglio F (1995). NMRPipe: a multidimensional spectral processing system based on UNIX pipes. J. Biomol. NMR.

[73] Orekhov VY, Jaravine VA (2011). Analysis of non-uniformly sampled spectra with multi-dimensional decomposition. Prog. Nucl. Magn. Reson. Spectrosc..

[74] Kazimierczuk K, Orekhov VY (2011). Accelerated NMR spectroscopy by using compressed sensing. Angew. Chem. Int. Ed..

[75] Vranken WF (2005). The CCPN data model for NMR spectroscopy: development of a software pipeline. Proteins.

