# Supplementary material for: Structural resolution of switchable states of a de novo peptide assembly
Source: Nat Commun. 2021 Mar 9;12:1530. doi: 10.1038/s41467-021-21851-8 (PMC7943578; doi:10.1038/s41467-021-21851-8)
Supplement: Supplementary file 1 — Supplementary Information [file 41467_2021_21851_MOESM1_ESM.pdf]

Supplementary Information

Structural resolution of switchable states of a *de novo* peptide assembly

William M. Dawson[1,†], Eric J. M. Lang[1,2,†], Guto G. Rhys[1,3,†], Kathryn L. Shelley[1,4,†], Christopher Williams[1,2], R. Leo Brady[4], Matthew P. Crump[1,2], Adrian J. Mulholland[1,2], and Derek N. Woolfson[1,2,4]*

[1]School of Chemistry, University of Bristol, Cantock's Close, Bristol, BS8 1TS, UK.

[2]BrisSynBio, University of Bristol, Life Sciences Building, Tyndall Avenue, Bristol BS8 1TQ, UK.

[3]Department of Biochemistry, University of Bayreuth, Universitätsstraße 30, 95447 Bayreuth, Germany.

[4]School of Biochemistry, University of Bristol, Medical Sciences Building, University Walk, Bristol, BS8 1TD, UK.

[†]These authors contributed equally to this work.

*Corresponding author. Email: d.n.woolfson@bristol.ac.uk

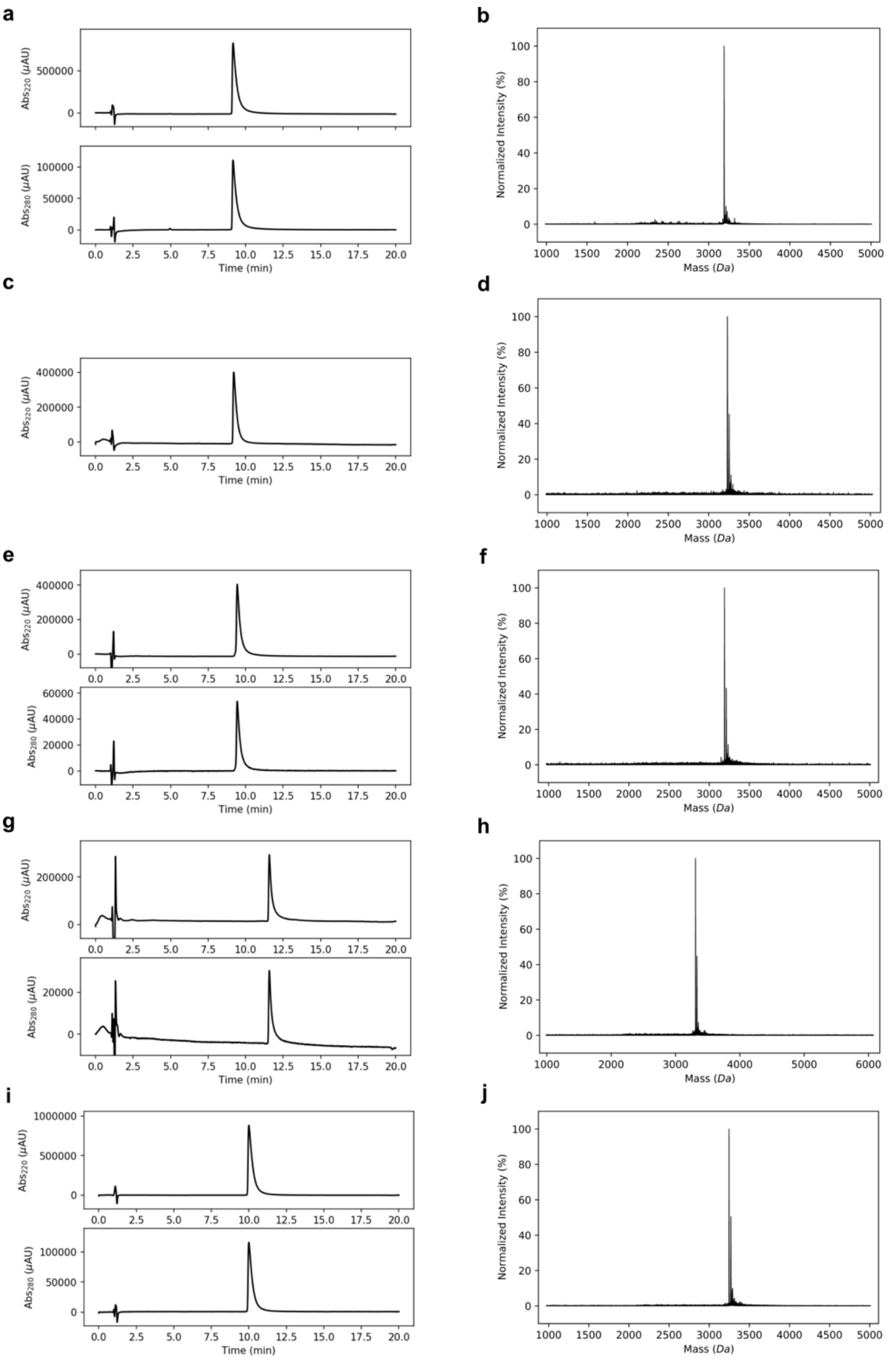

Supplementary Figure 1: Peptide characterisation data. Analytical HPLC monitored at 220 nm (left, top) and 280 nm (left, bottom) and MALDI-TOF (right) data. CC-Type2-($L_aI_dG_e$)$_4$ (**a** and **b**): calculated mass, 3189.8 Da [M+H]$^+$; observed mass, 3189.7 Da [M+H]$^+$. CC-Type2-($L_aI_dG_e$)$_4$-W19BrPhe (**c** and **d**): calculated mass, 3229.724 [M+H]$^+$; observed mass: 3228.717 [M+H]$^+$. CC-Type2-($L_aI_dG_e$)$_4$-[^{13}C-Ala$_{13}$] (**e** and **f**): calculated mass, 3190.828 Da [M+H]$^+$; observed mass, 3190.732 Da [M+H]$^+$. CC-Type2-($S_aL_dI_e$)$_4$-[^{13}C-Ala$_{18}$] (**g** and **h**): calculated mass, 3310.938 Da [M+H]$^+$; observed mass, 3310.681 Da [M+H]$^+$. CC-Type2-(L_aL_d)$_4$-[^{13}C-Ala$_{13}$] (**i** and **j**): calculated mass, 3246.891 Da [M+H]$^+$; observed mass: 3246.87 Da [M+H]$^+$.

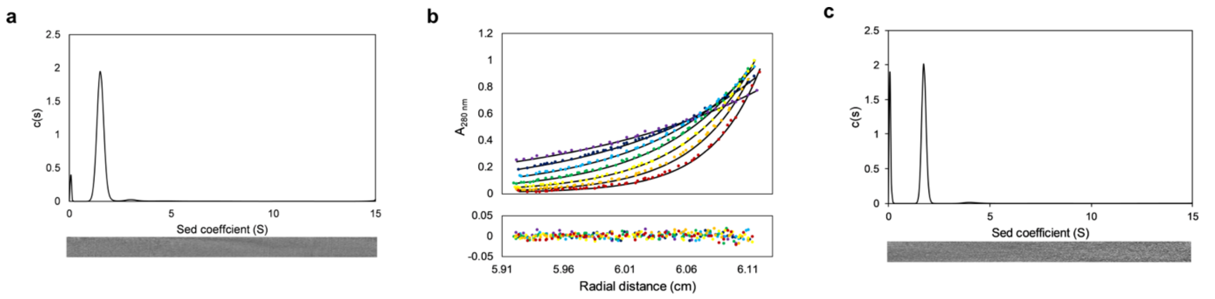

Supplementary Figure 2: Biophysical characterisation a: Sedimentation velocity (SV) AUC data for CC-Type2-($L_aI_dG_e$)$_4$ at 50 krpm ($\bar{v}$ = 0.771 $cm^3 g^{-1}$). Residuals are shown as a bitmap below the fitted data. Continuous c(s) distribution returned a molecular mass of 18136 Da corresponding to 5.7 x monomer mass at 95% confidence level (f/f_0 = 1.33, s = 1.526 S, $s_{20,w}$ = 1.563 S). **b:** Sedimentation equilibrium (SE) AUC data for CC-Type2-($L_aI_dG_e$)$_4$ between 15 and 33 krpm at 3 krpm intervals. Fitted single-ideal species model curves are overlaid in black and gave a molecular mass 17476 Da corresponding to 5.5 x monomer mass, 95% confidence limits 17394-17555 Da. Conditions: 150 μM and 70 μM peptide for SV and SE experiments respectively, PBS, pH 7.4, 20 °C. **c**: Sedimentation velocity (SV) AUC data for CC-Type2-($L_aI_dG_e$)$_4$ at 50 krpm ($\bar{v}$ = 0.773 $cm^3 g^{-1}$). Residuals are shown as a bitmap below the fitted data. Continuous c(s) distribution returned a molecular mass of 19796 Da corresponding to 6.2 x monomer mass at 95% confidence level (f/f_0 = 1.22, s = 1.755 S, $s_{20,w}$ = 1.784 S). Conditions: 200 μM peptide, 5 μM DPH, 5% DMSO v/v, PBS, pH 7.4, 25 °C.

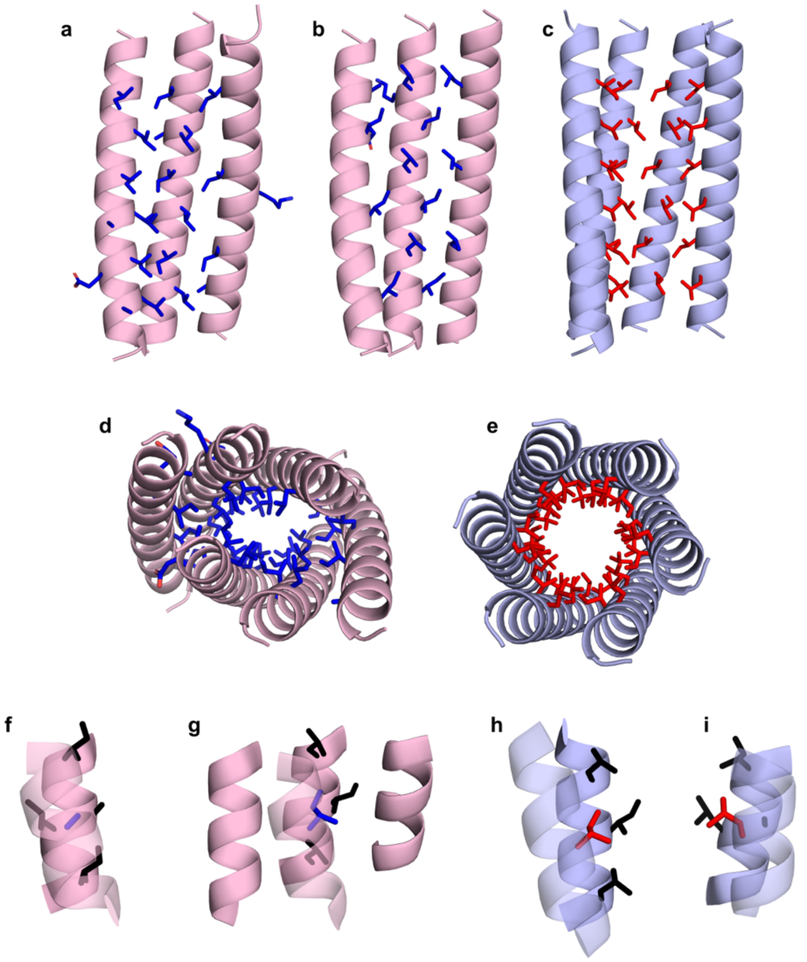

Supplementary Figure 3: Visualisation of iSOCKET outputs for the two states of CC-Type2-($L_aI_dG_e$)$_4$-W19BrPhe. **a**: Knob residues (blue) of the collapsed structure of CC-Type2-($L_aI_dG_e$)$_4$-W19BrPhe (red, chains G-I). **b**: Knob residues (blue) of the collapsed structure of CC-Type2-($L_aI_dG_e$)$_4$-W19BrPhe (red, chains J-L). **c**: Knob residues (red) of the open structure of CC-Type2-($L_aI_dG_e$)$_4$-W19BrPhe (blue, only chains A, B, E, F shown for clarity). **d** and **e**: Collapsed and open structure of CC-Type2-($L_aI_dG_e$)$_4$-W19BrPhe from above. **f** – **i**: Individual examples of KIH interactions from the closed (red) and open (blue) states. Knob residues are shown in blue or red, and the residues making up the hole shown in black. **f**, **g** and **i** are examples of KIH interactions with adjacent helices. **g** is an example of a KIH interaction with a non-adjacent helix.

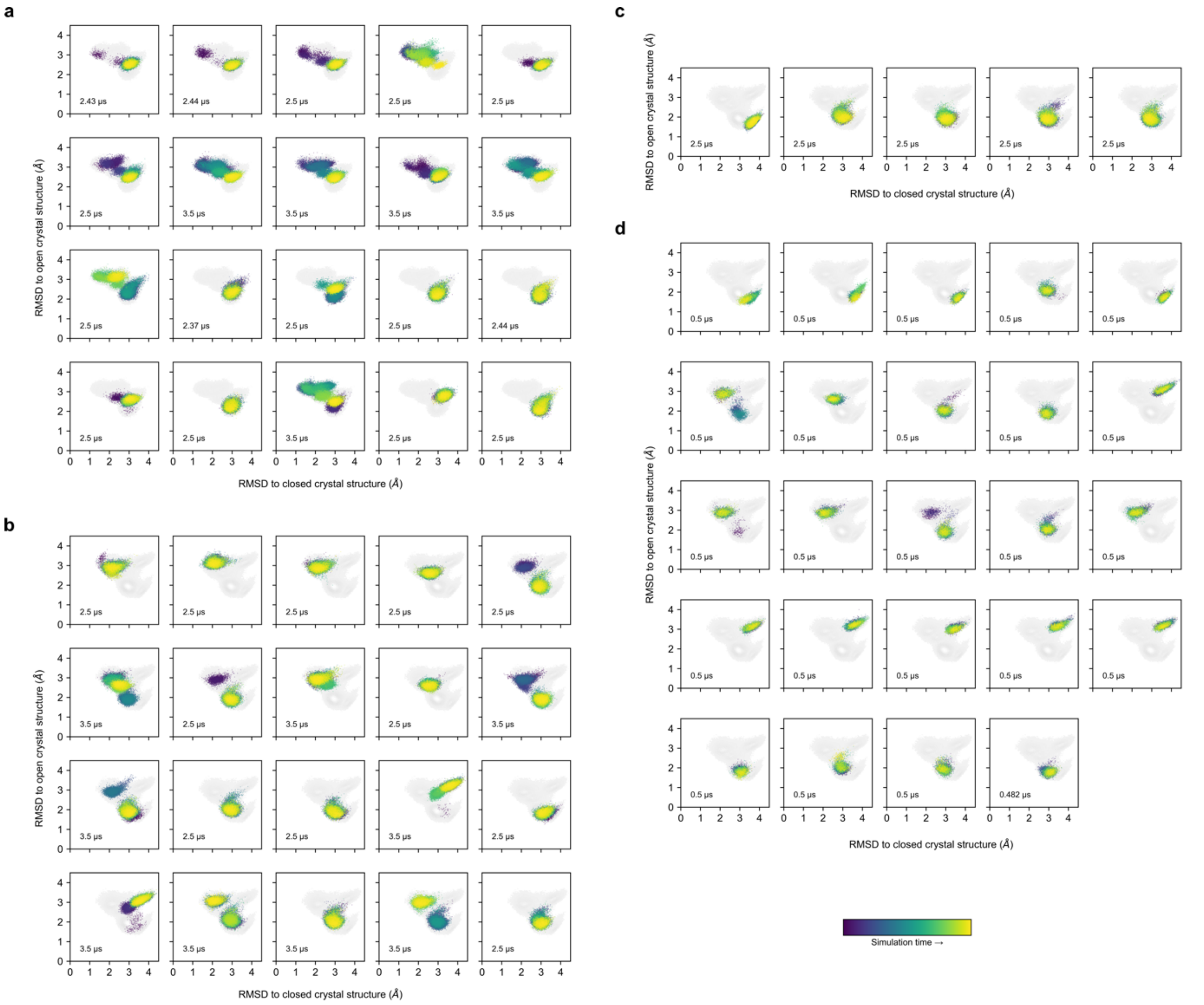

Supplementary Figure 4: Molecular dynamics. Projections along the RMSD coordinates of the ensemble of conformations sampled during each independent MD simulation. The different panels correspond to the simulations run in the absence of isopropanol (**a**), the initial simulations in the presence of isopropanol (**b**), the 5 additional simulations with isopropanol initiated from the open crystal structure (**c**), and the short simulations initiated from random conformations belonging to each core states in the presence of isopropanol (**d**) (see 1.8.3 for details). For **a** and **b** the top 10 plots correspond to the simulations started from the closed form whereas the bottom 10 plots correspond to the simulations started from the open crystal structures. The colour gradient from dark purple to yellow corresponds to an increasing simulation time, indicating when, during a simulation, specific regions of the conformational space were sampled. The relative free energy surfaces calculated for the ensemble of simulations in the absence and presence of isopropanol are shown with transparent light grey tones for reference.

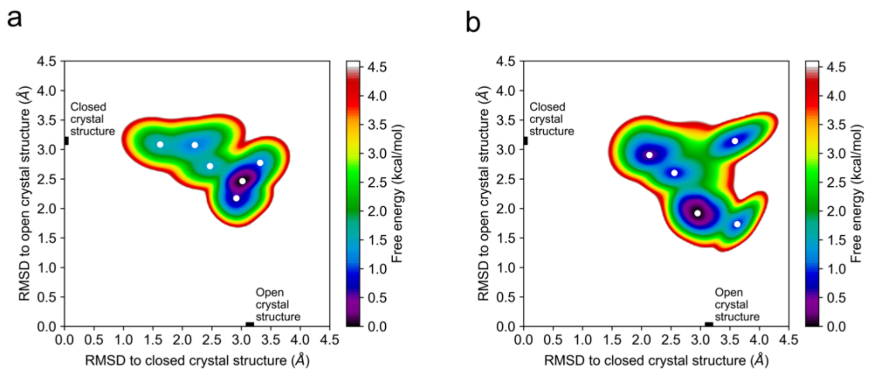

Supplementary Figure 5: Centres of the core states: Conformational free energy landscape calculated from the MD simulations in the absence (**a**) and presence (**b**) of isopropanol with the centres of each core state identified with InfleCS represented with white dots. We can see that those centres correspond to the free energy minima on the landscapes.

a

b

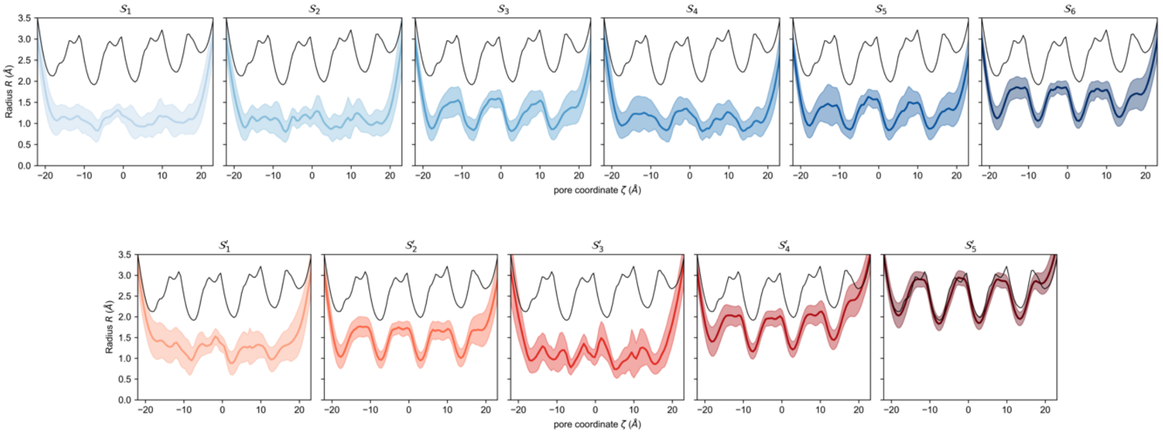

Supplementary Figure 6: Channel radii of the core state: Mean channel radii (thick lines) and standard deviation (transparent area) of the core states in the absence (**a**) and presence (**b**) of isopropanol. The thin black lines are for the radius of the open channel in the crystal structure.

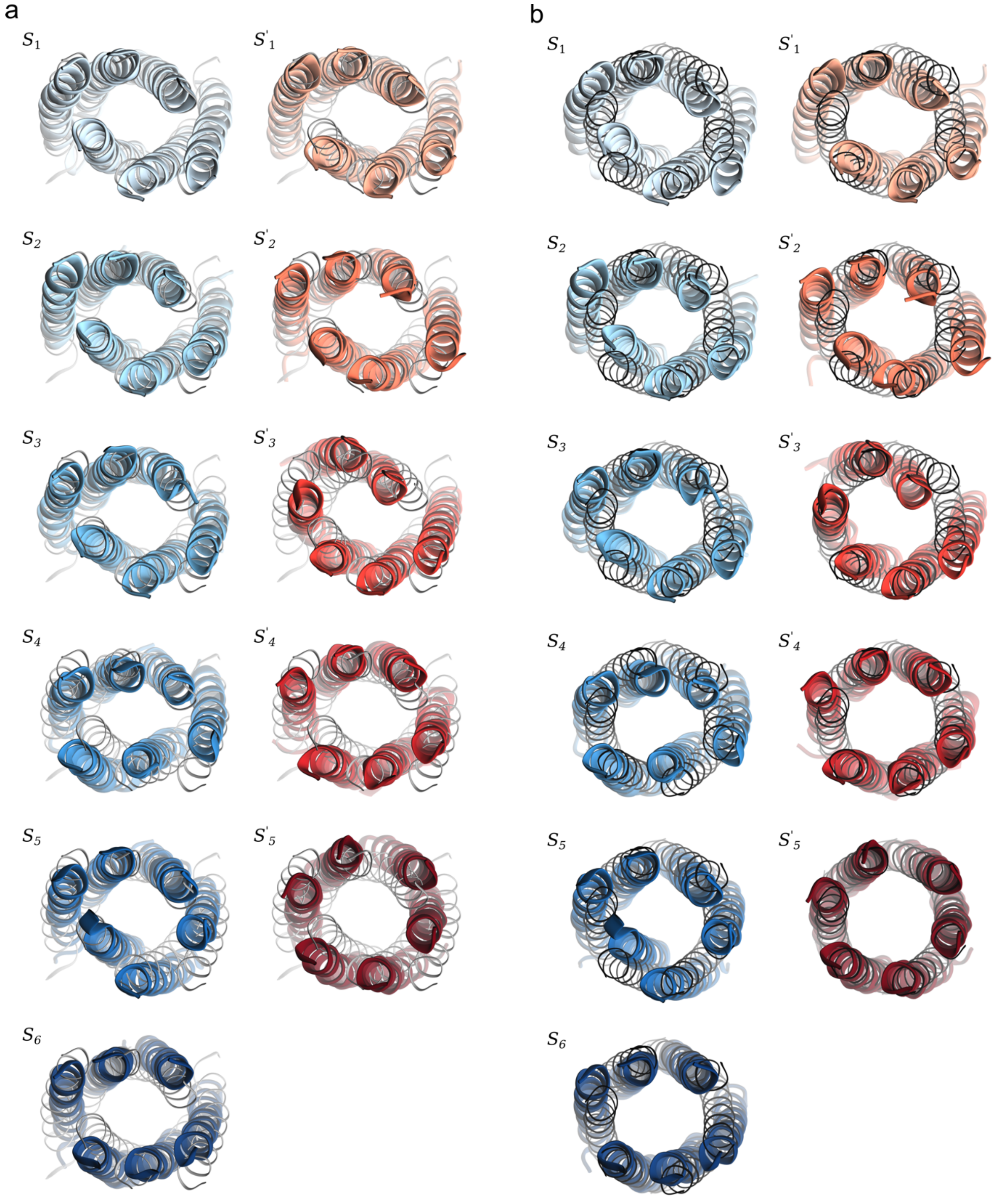

Supplementary Figure 7: Comparison of the core state with the X-ray crystal structures: Superimposition of a random structure taken from each core states with the closed (**a**) and open (**b**) X-ray crystal structures. The structures from the core states are shown as thick ribbons using the same colour code as elsewhere. The thin silver ribbon corresponds to the closed form crystal structure and the thin black ribbon to the open form crystal structure.

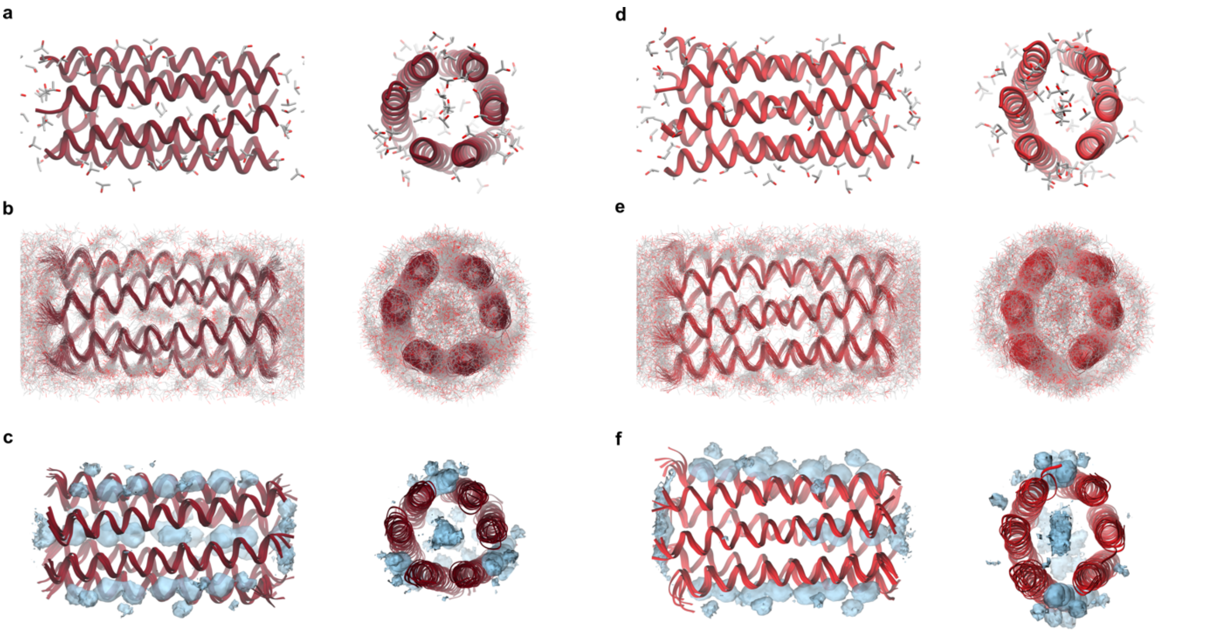

Supplementary Figure 8: Open metastable states. Contribution of isopropanol to the stabilisation of specific core states. Orthogonal views of structures belonging to $\mathcal{S}'_5$ (**a**, **b**, **c**), $\mathcal{S}'_4$ (**d**, **e**, **f**) depicting the position of isopropanol molecules (shown as sticks) around the peptide for a random single frame (**a**, **d**); the superimposition of isopropanol (shown as thin sticks) over 100 frames (**b**, **e**); and the isodensity of IPA (shown as transparent blue isosurface) at an isovalue of 2.75 (**c**, **f**). In the case of $\mathcal{S}'_5$, IPA molecules infiltrate the interface between every two helices and enter the lumen, stabilising the open state. For $\mathcal{S}'_4$, IPA interacts with at the interface of two trimers and is able to enter the end of the channel, stabilising a partially open state. Not all isopropanol binding sites are occupied at the same time (**a**, **d**) and individual isopropanol molecules tend to have a low residence time in a binding site (**b**, **e**) and to exchange rapidly with neighbouring or passing isopropanol molecules.

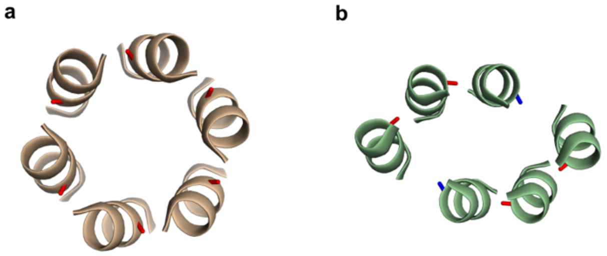

Supplementary Figure. 9: Labelled Ala residues a: Equivalent ^{13}C-Ala$_{18}$ environments (red) in CC-Type2-(S$_a$L$_d$I$_e$)$_4$ (PDB:4PN9). **b:** Three ^{13}C-Ala$_{13}$ environments (red and blue) in CC-Type2-(L$_a$L$_d$)$_4$ (PDB:6G6A). In the NMR experiments, we see two HSQC peaks with an intensity ratio of 2:1, and we assume that two of the Ala residues (in red) are in magnetically equivalent/similar environments.

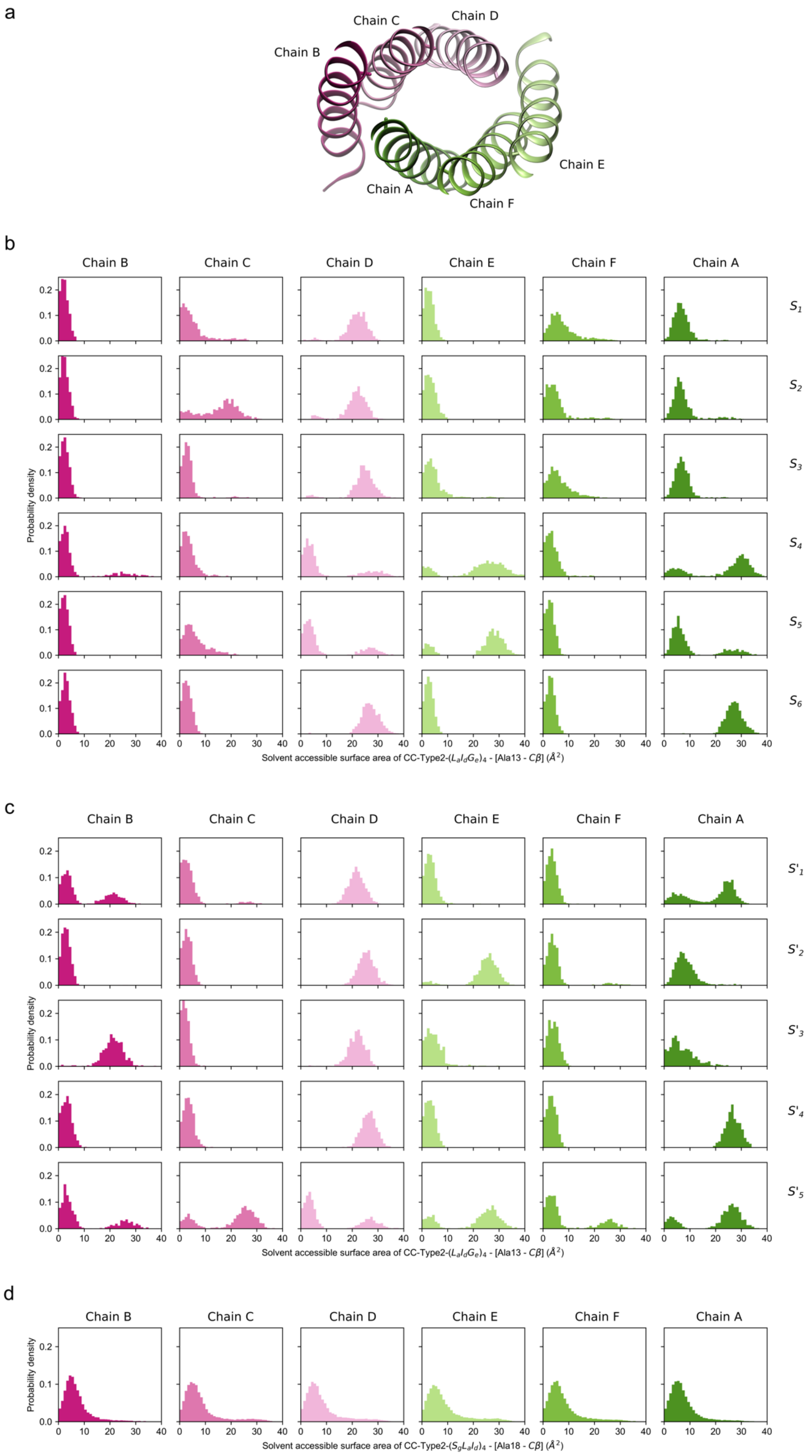
a
Chain C
Chain D
Chain B
Chain E
Chain A
Chain F
b
Chain B
Chain C
Chain D
Chain E
Chain F
Chain A
S1
S2
S3
S4
S5
S6
Probability density
Solvent accessible surface area of CC-Type2-(LaIdGe)4 - [Ala13 - Cβ] (Å2)
c
Chain B
Chain C
Chain D
Chain E
Chain F
Chain A
S'1
S'2
S'3
S'4
S'5
Probability density
Solvent accessible surface area of CC-Type2-(LaIdGe)4 - [Ala13 - Cβ] (Å2)
d
Chain B
Chain C
Chain D
Chain E
Chain F
Chain A
Probability density
Solvent accessible surface area of CC-Type2-(SgLaId)4 - [Ala18 - Cβ] (Å2)

Supplementary Figure 10: Solvent accessible surface area analysis of Ala-13 Cβ from the MD simulations. a: Structure showing chain IDs with respect to the closed form X-ray crystal structure. Solvent accessible surface area (SASA) of Ala-13 Cβ was calculated for each chain of each core state for the peptide conformations sampled during the simulations in water (**b**) and in the water:isopropanol mixture (**c**). **d**: SASA calculated for Ala-18 Cβ CC-Type2-($S_gL_aI_d$)$_4$ is shown for reference. Results suggest that Ala-13 Cβ is either buried (SASA < 10 Å^2) or exposed (SASA > 10 Å^2), and because of the chain symmetry, we expect the environment to be similar within each of those cases. By weighting the proportion of exposed versus buried Ala-13 Cβ for each state with the relative population of each state, we found that 26% of the environments classified as exposed and 74% as buried for the simulations in water (**b**). This is in complete agreement with the 1:3 ratio of the peaks observed by NMR. For the simulations in the presence of isopropanol, 36% of the environments classified as exposed, consistent with the more open states sampled in the co-solvent. However, we note that the SASA calculations were performed without isopropanol molecules present, which could affect interpretation of these data.

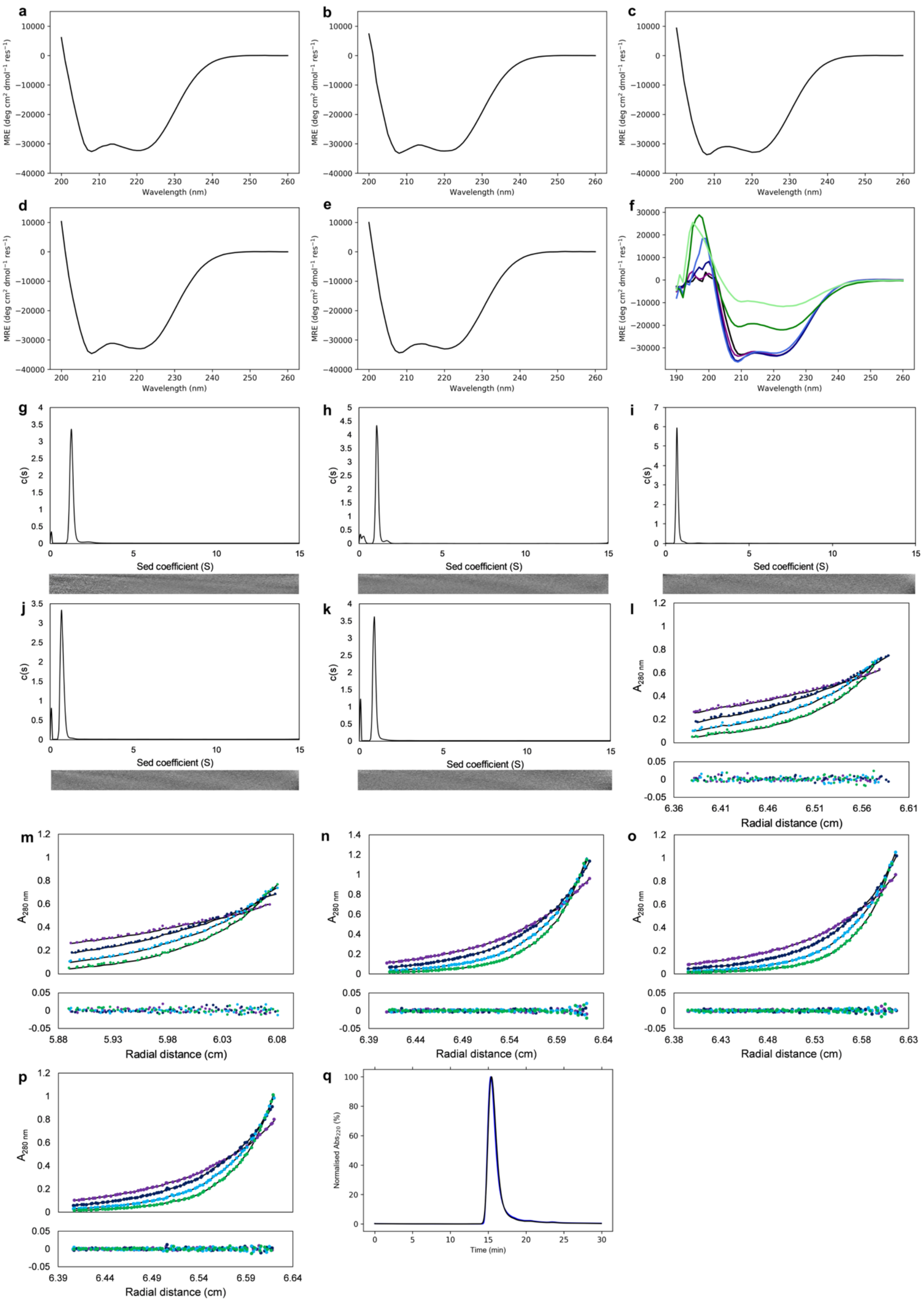
a
b
c
d
e
f
MRE (deg cm² dmol⁻¹ res⁻¹)
Wavelength (nm)
g
h
i
j
k
c(s)
Sed coefficient (S)
l
m
n
o
p
A280 nm
Radial distance (cm)
q
Normalised Abs220 (%)
Time (min)

Supplementary Figure 11: Further biophysical characterisation. a-f: CD spectra of CC-Type2-($L_aI_dG_e$)$_4$ in the presence of isopropanol (IPA). IPA was titrated in 5% v/v steps from 5% to 25% (**a** to **e,** respectively) and 10% v/v steps from 30% to 80% (**f**). Conditions: 300 μM peptide, 0-25% v/v IPA, PBS, pH 7.4, 20 °C. **g-k**: Sedimentation velocity (SV) AUC data for CC-Type2-($L_aI_dG_e$)$_4$ at 50 krpm ($\overline{v}$ = 0.773 cm^3 g^{-1}) in the presence of 5% to 25% v/v isopropanol (IPA) (**g** to **k**). Residuals are shown as a bitmap below the fitted data. **g**: Continuous c(s) distribution returned a molecular mass of 17680 Da corresponding to 5.5 x monomer mass (MM) at 95% confidence level (f/f_0 = 1.23, s = 1.301 S, $s_{20,w}$ = 1.656 S). **h**: Continuous c(s) distribution returned a molecular mass of 18230 Da corresponding to 5.7 x MM at 95% confidence level (f/f_0 = 1.19, s = 1.100 S, $s_{20,w}$ = 1.747 S). **i**: Continuous c(s) distribution returned a molecular mass of 17831 Da corresponding to 5.6 x MM at 95% confidence level (f/f_0 = 1.20, s = 0.914 S, $s_{20,w}$ = 1.719 S). **j**: Continuous c(s) distribution returned a molecular mass of 17830 Da corresponding to 5.6 x MM at 95% confidence level (f/f_0 = 1.31, s = 0.732 S, $s_{20,w}$ = 1.572 S). **k**: Continuous c(s) distribution returned a molecular mass of 17590 Da corresponding to 5.5 x MM at 95% confidence level (f/f_0 = 1.21, s = 0.707 S, $s_{20,w}$ = 1.687 S). Conditions: 150 μM peptide, 5-25% v/v IPA, PBS, pH 7.4, 20 °C. **k-p:** Sedimentation equilibrium (SE) AUC data for CC-Type2-($L_aI_dG_e$)$_4$ with 5% to 25% v/v isopropanol (IPA) (**l** to **p**) between 20 and 35 or 30 and 45 krpm at 5 krpm intervals. Fitted single-ideal species model curves are overlaid in black. **l**: 17560 Da corresponding to 5.5 x monomer mass (MM), 95% confidence limits 17280-17820 Da. **m**: 17600 Da corresponding to 5.5 x MM, 95% confidence limits 17390-17780 Da. **n**: 18320 Da corresponding to 5.7 x MM, 95% confidence limits 17770-18870 Da. **o**: 20040 Da corresponding to 6.3 x MM, 95% confidence limits 19540-20520 Da. **p**: 18040 Da corresponding to 5.7 x MM, 95% confidence limits 17540-18570 Da. Conditions: 70 μM peptide, 5-25% IPA, PBS, pH 7.4, 20 °C. **q**: Size exclusion chromatography for CC-Type2-($L_aI_dG_e$)$_4$ (blue) and CC-Type2-($L_aI_dG_e$)$_4$ (black) in 25% v/v isopropanol. Conditions: 150 μM peptide, 25% IPA, PBS, pH 7.4.

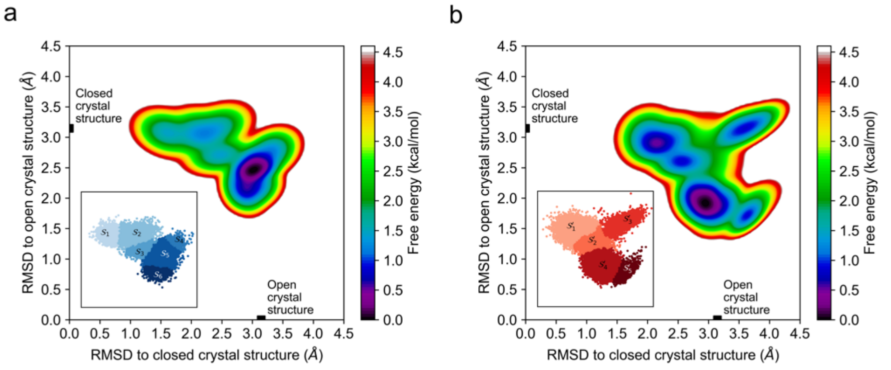

c

Population of the core states

Without IPA	With IPA
S_1: 5.6%	S'_1: 20.2%
S_2: 15.5%	S'_2: 9.4%
S_3: 4.6%	S'_3: 14.9%
S_4: 1.0%	S'_4: 49.3%
S_5: 58.2%	S'_5: 6.2%
S_6: 15.1%	

Supplementary Figure 12: Checking the convergence of the molecular dynamics simulations using a reduced dataset. a & b: Conformational free energy landscape calculated from the MD simulations as in Figure 3**,** in the absence (**a**) and presence (**b**) of isopropanol, but using a reduced dataset obtained by from only 1/50th of the total MD frames, randomly chosen. Regions for the core states identified with InfleCS are shown as insets. **c**: Estimated relative population for each core state for the reduced dataset. From this analysis, it is clear that both the free energy landscapes and their separation into core states are almost identical to what was obtained with the full data set and that the relative populations of the core state are very close to those obtained with the full dataset. We can therefore conclude to the convergence of the MD simulations for this region of the free energy landscape.

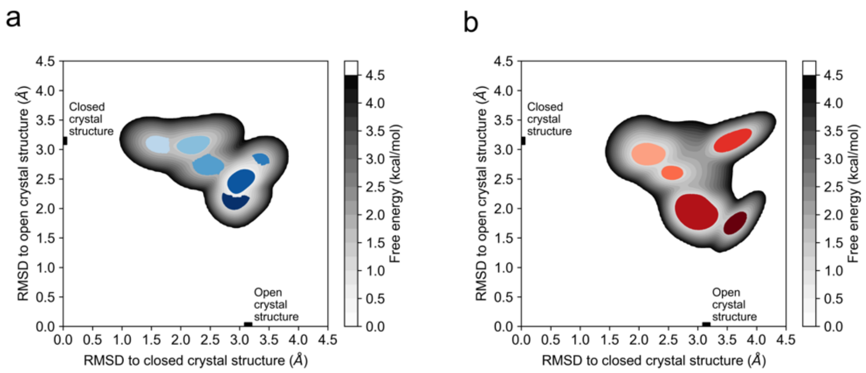

Supplementary Figure 13: Crisp clustering used for extracting representative structures: Conformational free energy landscape calculated from the MD simulations in the absence (**a**) and presence (**b**) of isopropanol with a "crisp" version of the clustering in which only the low energy conformation of each core states, and not the transition conformations, are retained. This approach was used to extract representative low energy conformations of each state for further analysis. For both plots, the free energy landscape is coloured using a grey gradient (the lighter the colour, the lower the energy), and the crisp clusters are identified with blue (**b**) or red (**a**) surfaces, using the same colour code used to identify states in Figure 3 of the main text.

Supplementary Table 1: Sequences of *de novo* peptides in this study.

Systematic Name	Heptad repeat (*gabcdef*)	Sequence
CC-Type2-$(L_aI_dG_e)_4$	ALKEIG*f*	Ac-GEIGQALKEIGKALKEIGWALKEIGQALKG-NH_2
CC-Type2-$(L_aI_dG_e)_4$-W19BrPhe	ALKEIG*f*	Ac-GEIGQALKEIGKALKEIGΦALKEIGQALKG-NH_2
CC-Type2-$(L_aI_dG_e)_4$-[^{13}C-Ala_{13}]	ALKEIG*f*	Ac-GEIGQALKEIGK**A**LKEIGWALKEIGQALKG-NH_2
CC-Type2-$(S_gL_aI_d)_4$-[^{13}C-Ala_{18}]	SLKEIA*f*	Ac-GEIAKSLKEIAKSLKEI**A**WSLKEIAKSLKG-NH_2
CC-Type2-$(L_aL_d)_4$-[^{13}C-Ala_{13}]	ALKELA*f*	Ac-GELAQALKELAK**A**LKELAWALKELAQALKG-NH_2

Φ = 4-bromo-phenylalanine

Position of the ^{13}C-isotopically labelled alanine residue is shown in bold

Supplementary Table 2: Merging and refinement statistics for CC-Type2-($L_aI_dG_e$)$_4$-W19BrPhe.

	**CC-Type2-($L_aI_dG_e$)$_4$-W19BrPhe**
Wavelength (Å)	0.91899
Resolution range (Å)	58.92-2.01 [58.92-8.99] (2.08-2.01)
Space group	P 21 21 2
Unit cell lengths (Å)	58.92 128.1 56.53
Unit cell angles (°)	90 90 90
Total reflections	37517 [3800] (38123)
Unique reflections	29226 [386] (2876)
Multiplicity	12.8 [9.8] (13.3)
Completeness (%)	99.9 [96.8] (99.9)
Mean I/sigma(I)	13.4 [29.8] (1.8)
Wilson B-factor Å^2)	37.11
R-merge(I)	0.136 [0.057] (1.638)
R-meas(I)	0.142 [0.060] (1.704)
R-pim	0.040 [0.019] (0.465)
CC1/2	0.998 (0.565)
CC*	1 (0.858)
Reflections used in refinement	29220 (2876)
Reflections used for R-free	1396 (134)
R-work	0.207 (0.275)
R-free	0.261 (0.308)
CC(work)	0.961 (0.786)
CC(free)	0.937 (0.748)
Number of non-hydrogen atoms	2806
macromolecules	2633
ligands	24
solvent	149
Protein residues	364
RMS(bonds)	0.007
RMS(angles)	1.04
Ramachandran favored (%)	99.66
Ramachandran allowed (%)	0
Ramachandran outliers (%)	0.34
Rotamer outliers (%)	0.87
Clashscore	3.72
Average B-factor	46.22
macromolecules	45.89
ligands	48.47
solvent	51.69
Number of TLS groups	12

Data in square brackets [ ] and parentheses ( ) represent statistical data related to inner and outer shells, respectively. Non bracketed data represent overall statistical data.

Supplementary Table 3: Helix-helix distances of central two heptads of the two states of CC-Type2-($L_aI_dG_e$)$_4$-W19BrPhe aligned by helix 1.

Backbone atom	Helix Translation Distance (Å) Helix 2	Helix 3	Helix 4	Helix 5	Helix 6
**1**	2.5	5	5.9	4.4	0.8
**2**	2.2	4.9	6.1	4.1	0.7
**3**	1.8	4.8	5.9	4.5	0.6
**4**	1.5	4.7	5.6	4.9	0.8
**5**	1.2	4.7	5.6	5.4	0.7
**6**	1.6	4.9	5.7	5.7	0.6
**7**	2	5.2	5.7	5.7	0.5
**8**	2.4	5.4	5.8	6	0.3
**9**	2.4	5.5	6.3	5.6	0.2
**10**	2.3	5.4	6.5	5.1	0.2
**11**	2.4	5.5	6.9	4.7	0.2
**12**	1.9	5.1	6.8	4.8	0.1
**13**	1.6	5.4	6.5	5	0.3
**14**	1.2	5.1	6.4	5.4	0.5
**15**	1.1	4.9	6.3	5.9	0.5
**16**	1.3	5	6.1	6.1	0.3
**17**	1.5	5.4	6.1	6.7	0.3
**18**	1.7	6	6.5	6.5	0.3
**19**	2.1	5.7	6.7	6	0.2
**20**	2.3	6.3	7.2	5.8	0.4
**21**	2	5.9	7.4	5.6	0.6
**22**	1.8	6.3	7.3	5.3	0.6
**23**	1.6	5.5	7.4	5.2	0.8
**24**	1.2	5.9	7.2	5.7	0.9
**25**	1.1	5.8	6.8	6.1	0.8
**26**	0.8	5.4	6.7	6.7	0.9
**27**	1.1	6.1	6.9	6.9	0.9
**28**	1.6	5.8	7	6.8	0.7
**29**	1.9	6.5	7.3	7.1	0.7
**30**	1.9	6.8	7.6	7.3	0.8
**31**	1.9	6.8	7.8	6.3	0.9
**32**	1.9	7.1	8.1	5.9	1.1
**33**	1.5	6.4	8.1	6	1.2
**34**	1.1	6.9	7.9	6.2	1.2
**35**	0.9	6.4	7.9	6.5	1.4
**36**	0.7	6.2	7.8	7.2	1.3
**37**	0.8	6.3	7.6	7.5	1.1
**38**	0.8	6.6	7.6	8	1
**39**	0.8	7.5	8	7.9	1
**40**	1.1	7.2	8.2	7.4	1
**41**	1.4	8.1	8.8	7.3	1.3
**42**	1.3	8.1	9	7	1.5
**Average**	**1.58 ± 0.52**	**5.92 ± 0.89**	**6.98 ± 0.89**	**6.05 ± 0.87**	**0.72 ± 0.37**

Supplementary Table 4: Simplified iSOCKET output for the αHB structure of CC-Type2-($L_aI_dG_e$)$_4$-W19BrPhe.

Knob		Hole 1		Hole 2		Hole 3		Hole 4	
**Helix**	**Residue**	**Helix**	**Residue**	**Helix**	**Residue**	**Helix**	**Residue**	**Helix**	**Residue**
**A**	**10**	B	7	B	10	B	11	B	14
**A**	**17**	B	14	B	17	B	18	B	21
**A**	**24**	B	21	B	24	B	25	B	28
**A**	**7**	F	3	F	6	F	7	F	10
**A**	**14**	F	10	F	13	F	14	F	17
**A**	**21**	F	17	F	20	F	21	F	24
**B**	**7**	A	3	A	6	A	7	A	10
**B**	**14**	A	10	A	13	A	14	A	17
**B**	**21**	A	17	A	20	A	21	A	24
**B**	**10**	C	7	C	10	C	11	C	14
**B**	**17**	C	14	C	17	C	18	C	21
**B**	**24**	C	21	C	24	C	25	C	28
**C**	**7**	B	3	B	6	B	7	B	10
**C**	**14**	B	10	B	13	B	14	B	17
**C**	**21**	B	17	B	20	B	21	B	24
**C**	**10**	D	7	D	10	D	11	D	14
**C**	**17**	D	14	D	17	D	18	D	21
**C**	**24**	D	21	D	24	D	25	D	28
**D**	**7**	C	3	C	6	C	7	C	10
**D**	**14**	C	10	C	13	C	14	C	17
**D**	**21**	C	17	C	20	C	21	C	24
**D**	**10**	E	7	E	10	E	11	E	14
**D**	**17**	E	14	E	17	E	18	E	21
**E**	**7**	D	3	D	6	D	7	D	10
**E**	**14**	D	10	D	13	D	14	D	17
**E**	**21**	D	17	D	20	D	21	D	24
**E**	**10**	F	7	F	10	F	11	F	14
**E**	**17**	F	14	F	17	F	18	F	21
**E**	**24**	F	21	F	24	F	25	F	28
**F**	**10**	A	7	A	10	A	11	A	14
**F**	**17**	A	14	A	17	A	18	A	21
**F**	**24**	A	21	A	24	A	25	A	28
**F**	**7**	E	3	E	6	E	7	E	10
**F**	**14**	E	10	E	13	E	14	E	17
**F**	**21**	E	17	E	20	E	21	E	24

Supplementary Table 5: iSOCKET output for the collapsed structure of CC-Type2-($L_aI_dG_e$)$_4$-W19BrPhe.

Knob		Hole 1		Hole 2		Hole 3		Hole 4	
**Helix**	**Residue**	**Helix**	**Residue**	**Helix**	**Residue**	**Helix**	**Residue**	**Helix**	**Residue**
**G**	**7**	H	3	H	6	H	7	H	10
**G**	**14**	H	10	H	13	H	14	H	17
**G**	**21**	H	17	H	20	H	21	H	24
**G**	**25**	H	20	H	23	H	24	H	27
**G**	**10**	K	7	K	10	K	11	K	14
**G**	**17**	K	14	K	17	K	18	K	21
**G**	**24**	K	21	K	24	K	25	K	28
**G**	**6**	L	3	L	6	L	7	L	10
**G**	**9**	L	7	L	10	L	11	L	14
**G**	**13**	L	10	L	13	L	14	L	17
**H**	**10**	G	7	G	10	G	11	G	14
**H**	**13**	G	11	G	14	G	15	G	18
**H**	**17**	G	14	G	17	G	18	G	21
**H**	**20**	G	18	G	21	G	22	G	25
**H**	**24**	G	21	G	24	G	25	G	28
**H**	**7**	I	3	I	6	I	7	I	10
**H**	**11**	I	6	I	9	I	10	I	13
**H**	**14**	I	10	I	13	I	14	I	17
**H**	**21**	I	17	I	20	I	21	I	24
**I**	**6**	H	4	H	7	H	8	H	11
**I**	**10**	H	7	H	10	H	11	H	14
**I**	**17**	H	14	H	17	H	18	H	21
**I**	**24**	H	21	H	24	H	25	H	28
**I**	**14**	J	9	J	10	J	12	J	13
**J**	**7**	K	3	K	6	K	7	K	10
**J**	**14**	K	10	K	13	K	14	K	17
**J**	**21**	K	17	K	20	K	21	K	24
**K**	**7**	G	3	G	6	G	7	G	10
**K**	**14**	G	10	G	13	G	14	G	17
**K**	**21**	G	17	G	20	G	21	G	24
**K**	**10**	J	7	J	10	J	11	J	14
**K**	**17**	J	14	J	17	J	18	J	21
**K**	**24**	J	21	J	24	J	25	J	28
**K**	**8**	L	2	L	5	L	6	L	9
**K**	**11**	L	6	L	9	L	10	L	13
**K**	**15**	L	9	L	12	L	13	L	16
**K**	**21**	L	17	L	20	L	21	L	24
**K**	**25**	L	23	L	24	L	27	L	28
**L**	**10**	G	6	G	9	G	10	G	13
**L**	**6**	K	4	K	7	K	8	K	11
**L**	**9**	K	8	K	11	K	12	K	15
**L**	**16**	K	15	K	18	K	19	K	22
**L**	**17**	K	14	K	17	K	18	K	21
**L**	**24**	K	21	K	24	K	25	K	28

Supplementary Table 6: Raw peak heights for CC-Type2-($L_aI_dG_e$)$_4$-[^{13}C-Ala$_{13}$], CC-Type2-(L_aL_d)$_4$-[^{13}C-Ala$_{13}$] and CC-Type2-($S_gL_aI_d$)$_4$-[^{13}C-Ala$_{18}$].

CC-Type2-($L_aI_dG_e$)$_4$-[^{13}C-Ala$_{13}$] peak heights (x10^6)									
**Peak (^{1}H-^{13}C)** **0% IPA**	1.44-18.8 ppm			1.44-18.4 ppm					
**15% IPA**	1.41-18.8 ppm			1.41-18.4 ppm			1.47-18.7 ppm		
**25% IPA**				1.41-18.5 ppm			1.49-18.8 ppm		
**IPA% (v/v)**	**Run 1**	**Run 2**	**Run 3**	**Run 1**	**Run 2**	**Run 3**	**Run 1**	**Run 2**	**Run 3**
**0**	16.6	18.5	18.9	4.91	6.33	5.67	-	-	
**5**	16.2	18.8	16.9	5.81	7.03	5.75	-	--	
**10**	11.7	13.2	12.1	6.15	7.50	6.12	-	-	
**15**	8.86	9.79	8.64	3.83	6.99	4.91	1.88	1.64	1.87
**20**	2.27	2.83	3.73	3.69	5.76	4.37	6.62	6.00	4.78
**25**	-	1.44	1.43	2.42	4.28	3.69	7.41	6.56	6.56

CC-Type2-(L_aL_d)$_4$-[^{13}C-Ala$_{13}$] peak heights (x10^6)						
**Peak (^{1}H-^{13}C)** **0% IPA**	1.47-18.2 ppm			1.65-20.0 ppm		
**25% IPA**	1.43-18.0 ppm			1.63-20.0 ppm		
**IPA% (v/v)**	**Run 1**	**Run 2**	**Run 3**	**Run 1**	**Run 2**	**Run 3**
**0**	3.68	4.54	7.19	6.83	8.24	10.5
**5**	3.20	3.14	3.86	5.59	5.82	7.34
**10**	3.04	4.53	5.04	5.46	5.94	6.64
**15**	3.04	3.83	4.72	4.92	5.51	7.10
**20**	2.73	3.56	3.60	4.07	4.08	4.72
**25**	1.53	2.04	2.14	2.29	2.35	3.29

CC-Type2-($S_gL_aI_d$)$_4$-[^{13}C-Ala$_{18}$] peak heights (x10^6)			
**Peak (^{1}H-^{13}C)** **0% IPA**	1.64-17.8 ppm		
**25% IPA**	1.62-17.9 ppm		
**IPA% (v/v)**	**Run 1**	**Run 2**	**Run 3**
**0**	9.58	6.43	7.51
**5**	10.0	9.40	7.98
**10**	10.9	13.3	9.45
**15**	13.0	11.4	8.51
**20**	9.49	8.32	8.22
**25**	6.71	7.39	6.11
