# Supplementary material for: Structural resolution of switchable states of a de novo peptide assembly
Source: Nat Commun. 2021 Mar 9;12:1530. doi: 10.1038/s41467-021-21851-8 (PMC7943578; doi:10.1038/s41467-021-21851-8)
Supplement: Supplementary file 3 — Reporting Summary [file 41467_2021_21851_MOESM3_ESM.pdf]

nature research

Corresponding author(s): Derek N. Woolfson

Last updated by author(s): Feb 3, 2021

Reporting Summary

Nature Research wishes to improve the reproducibility of the work that we publish. This form provides structure for consistency and transparency in reporting. For further information on Nature Research policies, see our Editorial Policies and the Editorial Policy Checklist.

Statistics

For all statistical analyses, confirm that the following items are present in the figure legend, table legend, main text, or Methods section.

n/a	Confirmed	
☐	☒	The exact sample size (*n*) for each experimental group/condition, given as a discrete number and unit of measurement
☐	☒	A statement on whether measurements were taken from distinct samples or whether the same sample was measured repeatedly
☒	☐	The statistical test(s) used AND whether they are one- or two-sided<br>*Only common tests should be described solely by name; describe more complex techniques in the Methods section.*
☒	☐	A description of all covariates tested
☒	☐	A description of any assumptions or corrections, such as tests of normality and adjustment for multiple comparisons
☐	☒	A full description of the statistical parameters including central tendency (e.g. means) or other basic estimates (e.g. regression coefficient) AND variation (e.g. standard deviation) or associated estimates of uncertainty (e.g. confidence intervals)
☒	☐	For null hypothesis testing, the test statistic (e.g. *F*, *t*, *r*) with confidence intervals, effect sizes, degrees of freedom and *P* value noted<br>*Give P values as exact values whenever suitable.*
☒	☐	For Bayesian analysis, information on the choice of priors and Markov chain Monte Carlo settings
☒	☐	For hierarchical and complex designs, identification of the appropriate level for tests and full reporting of outcomes
☒	☐	Estimates of effect sizes (e.g. Cohen's *d*, Pearson's *r*), indicating how they were calculated

Our web collection on statistics for biologists contains articles on many of the points above.

Software and code

Policy information about availability of computer code

Data collection	Circular diochrism: Spectra Manager (1.55). Size exclusion chromatography: ChromNav (2.0). Ligand binding: Clariostar (5.21 R2). Analytical ultracentrifugation: ProteomeLab XL-A (5.5). Molecular dynamics: Modeller (9.20), Schrodinger's Maestro (2018.3), Solvate 1.0, tleap from AmberTools 17, pmemb from Amber16 and Amber18. NMR: NMRPipe (10.9 Rev 2020.119.13.27), qMDD (3.2)
Data analysis	Ligand binding - SigmaPlot13.0. Analytical ultracentrifugation - SEDFIT (v15.2b)/SEDPHAT (v15.2b). X-ray crystallography - XIA2 (0.5.340-g5578c4a7-dials-1.6) pipeline (utilising AIMLESS (0.5.32), POINTLESS (1.11.1), XDS (Nov 1, 2016) and ctruncate (1.17.25)), Big EP automated pipeline (utilising Crank (2.0.148)), COOT (0.8.8), Phenix (1.10.1_2155), PyMOL (v1.3). Molecular dynamics - pytraj and cpptraj from AmberTools 19, PyEMMA (2.5.7), HOLE (2.2.005), VMD (1.9.5), InfleCS (1.0), NMR - CCPNMR Analysis (2.4.2)

For manuscripts utilizing custom algorithms or software that are central to the research but not yet described in published literature, software must be made available to editors and reviewers. We strongly encourage code deposition in a community repository (e.g. GitHub). See the Nature Research guidelines for submitting code & software for further information.

Data

Policy information about availability of data

All manuscripts must include a data availability statement. This statement should provide the following information, where applicable:

- Accession codes, unique identifiers, or web links for publicly available datasets
- A list of figures that have associated raw data
- A description of any restrictions on data availability

The coordinate and structure factor files for CC-Type2-(LaIdGe)4 have been deposited in the Protein Data Bank with accession code 6ZT1 (https://doi.org/10.2210/pdb6ZT1/pdb). Source data are provided with this paper. Script and compressed data files for the MD simulations are available at https://github.com/eric-jm-lang/MD-switch-paper. All other data presented in the study is available from the corresponding author on request.

Field-specific reporting

Please select the one below that is the best fit for your research. If you are not sure, read the appropriate sections before making your selection.

- [x] Life sciences
- [ ] Behavioural & social sciences
- [ ] Ecological, evolutionary & environmental sciences

For a reference copy of the document with all sections, see nature.com/documents/nr-reporting-summary-flat.pdf

Life sciences study design

All studies must disclose on these points even when the disclosure is negative.

Sample size	This is not a study where a hypothesis is tested through a statistical analysis of the results/observations of individuals. Therefore, issues relevant to statistical hypothesis testing such as sample size do not apply to the experimental data. For the molecular dynamics simulations, a total of 834825 conformations for the simulations with IPA, and 546868 conformations for the simulations without IPA, were generated.
Data exclusions	No experimental data was excluded from the study. For molecular dynamics simulations, conformations generated during the minimisation, heating and equilibration phase were not included in the analysis. Full details are in the "Methods" section
Replication	All attempts at replication were successful. CD spectra were measured 8 times and averaged. AUC-SV experiments were performed once for each experimental condition. AUC-SE experiments were performed on 3 samples for each experimental condition. Ligand binding assays were replicated 5 times. NMR experiments were replicated 3 times. Replication is not relevant for molecular dynamics simulations, as all runs were initiated with a different random velocity assignment and run on different GPUs, therefore every run was slightly different, which is desirable in order to better sample the conformational energy landscape.
Randomization	This is not a study where a hypothesis is tested through a statistical analyses of the results/observations of individuals. Therefore, issues relevant to statistical hypothesis testing such as randomization do not apply the experimental data. For molecular dynamics simulations, the starting velocities for the simulations depends on a pseudo random number generator and the seed used for it was based on the time and date of the simulation run, ensuring that the initial velocity distribution was always random and different for every run.
Blinding	This is not a study where a hypothesis is tested through a statistical analyses of the results/observations of individuals. Therefore, issues relevant to statistical hypothesis testing such as blinding to do not apply in this study.

Reporting for specific materials, systems and methods

We require information from authors about some types of materials, experimental systems and methods used in many studies. Here, indicate whether each material, system or method listed is relevant to your study. If you are not sure if a list item applies to your research, read the appropriate section before selecting a response.

Materials & experimental systems

n/a	Involved in the study
☒	☐ Antibodies
☒	☐ Eukaryotic cell lines
☒	☐ Palaeontology and archaeology
☒	☐ Animals and other organisms
☒	☐ Human research participants
☒	☐ Clinical data
☒	☐ Dual use research of concern

Methods

n/a	Involved in the study
☒	☐ ChIP-seq
☒	☐ Flow cytometry
☒	☐ MRI-based neuroimaging
