# Supplementary material for: Structural resolution of switchable states of a de novo peptide assembly
Source: Nat Commun. 2021 Mar 9;12:1530. doi: 10.1038/s41467-021-21851-8 (PMC7943578; doi:10.1038/s41467-021-21851-8)
Supplement: Supplementary file 2 — Peer Review File [file 41467_2021_21851_MOESM2_ESM.pdf]

Editorial Note: This manuscript has been previously reviewed at another journal that is not operating a transparent peer review scheme. This document only contains reviewer comments and rebuttal letters for versions considered at *Nature Communications*.

REVIEWERS' COMMENTS

Reviewer #2 (Remarks to the Author):

This is a revised version of a paper that I have previously reviewed for NCB. The authors have done a great job of revising the manuscript, and I believe that the simpler InfleCS clustering is cleaner than the MSM analysis presented originally. [Incidentally, I still find the question of exchange rates very interesting to understand, but realize that an in depth analysis of this would be well beyond this current work]. I am still not 100% convinced that the two states (or something very similar) seen in the crystal are sampled in solution, but I nonetheless agree that this is a very solid step forward, and that the data presented certainly points to an interesting and dynamic system. Congrats to the authors for a very interesting piece of work.
